# Unusual Length Dependence of the Conductance in Cumulene Molecular Wires

**DOI:** 10.1002/anie.201901228

**Published:** 2019-05-17

**Authors:** Wenjun Xu, Edmund Leary, Songjun Hou, Sara Sangtarash, M. Teresa González, Gabino Rubio‐Bollinger, Qingqing Wu, Hatef Sadeghi, Lara Tejerina, Kirsten E. Christensen, Nicolás Agraït, Simon J. Higgins, Colin J. Lambert, Richard J. Nichols, Harry L. Anderson

**Affiliations:** ^1^ Department of Chemistry University of Oxford Chemistry Research Laboratory Oxford OX1 3TA UK; ^2^ Department of Chemistry Donnan and Robert Robinson Laboratories University of Liverpool Liverpool L69 7ZD UK; ^3^ Surface Science Research Centre University of Liverpool Oxford Street Liverpool L69 3BX UK; ^4^ Department of Physics Lancaster University Lancaster LA1 4YW UK; ^5^ Instituto Madrileño de Estudios Avanzados (IMDEA) Calle Faraday 9, Campus Universitario de Cantoblanco 28049 Madrid Spain; ^6^ Departamento de Física de la Materia Condensada IFIMAC and Instituto “Nicolás Cabrera” Universidad Autónoma de Madrid 28049 Madrid Spain

**Keywords:** break junctions, conductance, cumulenes, molecular wires, single-molecule studies

## Abstract

Cumulenes are sometimes described as “metallic” because an infinitely long cumulene would have the band structure of a metal. Herein, we report the single‐molecule conductance of a series of cumulenes and cumulene analogues, where the number of consecutive C=C bonds in the core is *n*=1, 2, 3, and 5. The [*n*]cumulenes with *n*=3 and 5 have almost the same conductance, and they are both more conductive than the alkene (*n*=1). This is remarkable because molecular conductance normally falls exponentially with length. The conductance of the allene (*n*=2) is much lower, because of its twisted geometry. Computational simulations predict a similar trend to the experimental results and indicate that the low conductance of the allene is a general feature of [*n*]cumulenes where *n* is even. The lack of length dependence in the conductance of [3] and [5]cumulenes is attributed to the strong decrease in the HOMO–LUMO gap with increasing length.

Long molecules generally conduct electricity less well than short ones, and this can be a problem when designing molecular wires for mediating efficient charge transport over distances of several nanometers. When a homologous series of oligomers are connected between metal electrodes, and the transport mechanism is coherent tunneling, the conductance *G* of each oligomer typically decreases exponentially with its molecular length *L* according to Equation (1),^1^

(1) $$G\propto e^{-\beta L}$$

where *β* is the exponential attenuation factor, which is normally in the range of 0.2–0.5 Å^−1^ for a conjugated organic π‐system.^2^ It has been predicted that molecules with low bond length alternation (BLA) will give unusual attenuation factors, such as *β*≈0 (i.e., conductance independent of length) or even *β*<0 (i.e., conductance increasing with length).^3^, ^4^ Cumulenes are the simplest type of neutral π‐system not to exhibit substantial BLA.^5^, ^6^, ^7^ Herein, we report an experimental and computational investigation of the length dependence of charge transport through these linear carbon chains. Recently, near length‐independent conductances were reported for a set of cyanine dyes, which constitute a class of cationic π‐systems with BLA≈0.^8^

Cumulenes and polyynes are the two types of linear chains of sp‐hybridized carbon atoms: In cumulenes, the carbon atoms are linked by double bonds, whereas in polyynes, there are alternating single and triple bonds (Figure 1). Cumulenes and polyynes have fascinated chemists for many years as models for carbyne, the infinite 1D form of carbon.^5^, ^6^ Cumulenes are said to have a “metallic” electronic structure,^4^, ^5^, ^6^, ^7^, ^9^, ^10^, ^11^ because an infinitely long cumulene would have a band structure characteristic of a metal, with a partially occupied band derived from the π and π* orbitals. In contrast polyynes have a π–π* gap that persists even in long chains, and an infinite polyyne is expected to be a semiconductor.^10^, ^12^ This difference in electronic structure is a direct consequence of the different BLAs. Although there is some BLA in cumulenes,^7^ it is much more subtle than the alternation between short C≡C and long C−C bonds in polyynes.

**Figure 1:**
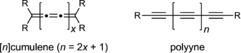
Cumulenes and polyynes: Two types of linear sp carbon chains.

Here, we report an experimental investigation of the conductances of the family of cumulenes **1**, **2**, **3**, and **5** shown in Figure 2. The [4]cumulene **4** is included in our theoretical investigation, but has not yet been tested experimentally. All of these molecules have two terminal 4‐thioanisole substituents for binding to gold electrodes. Single‐molecule conductances were measured using the scanning tunneling microscopy break junction (STM‐BJ) method, using a gold tip and a gold surface,^13^ as described in detail previously.2d

**Figure 2:**
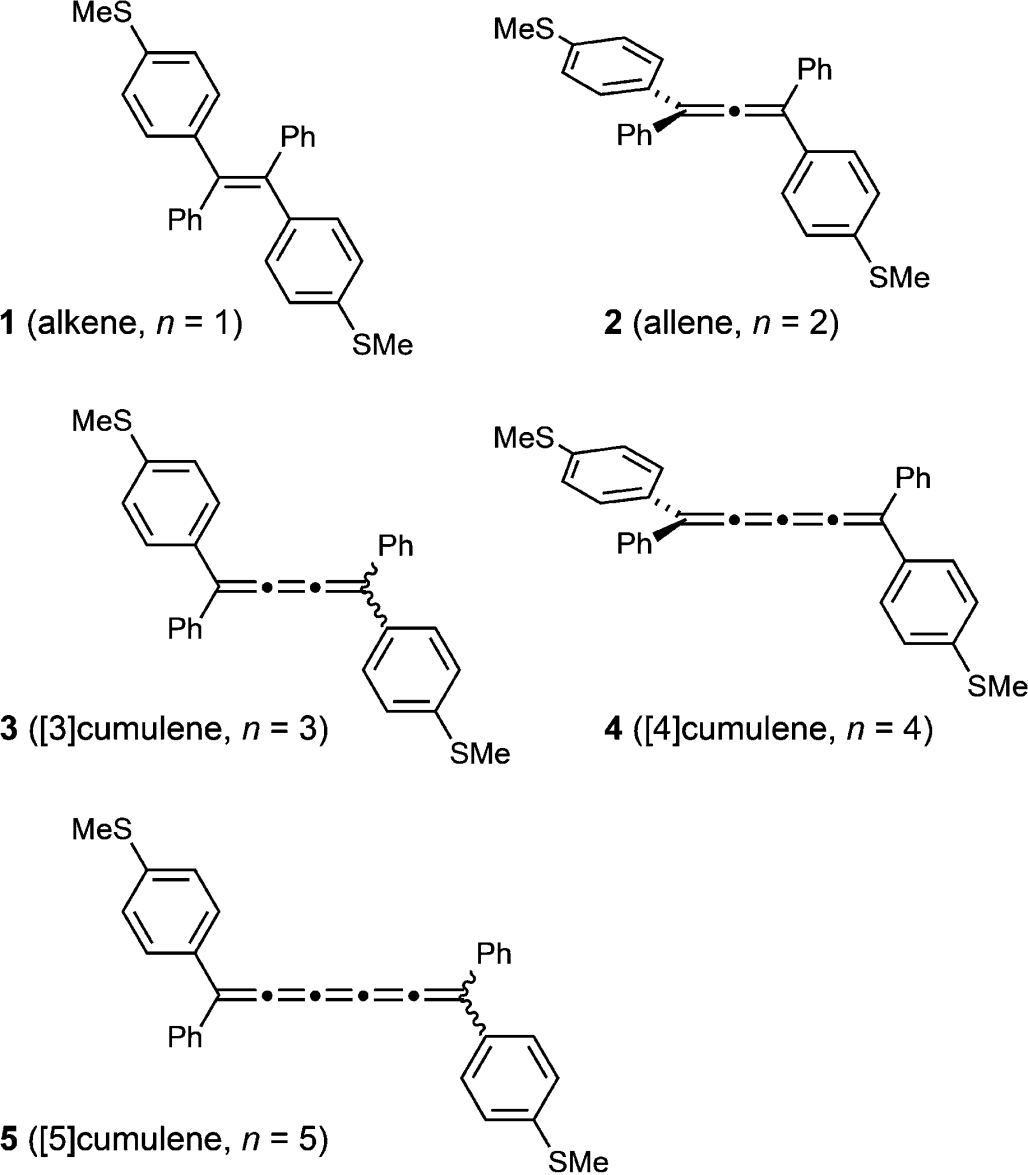
Structures of compounds **1**–**5**. (Note that only **1**, **2**, **3**, and **5** have been tested experimentally.)

Compounds **1**, **2**, **3**, and **5** were synthesized as described in the Supporting Information.^14^ The alkene **1** was recrystallized to give the *E* isomer, as confirmed by single‐crystal X‐ray diffraction.^15^ Allene **2** is racemic. Cumulenes **3** and **5** are 1:1 mixtures of the *E* and *Z* isomers (as confirmed by ^1^H NMR spectroscopy); we were unable to separate these stereoisomers. We expect them to interconvert readily under ambient conditions,^16^ and to have similar conductances (see the Supporting Information, Figure S7).

The experimental conductance results for compounds **1**–**3** and **5** are summarized in Figure 3 and Table 1. For reference, we have also measured 4,4′‐bis(methylthio)biphenyl, which has no double bonds between the phenyl rings (Section S3.7). The 2D histograms (Figure 3 a–d) show how the conductance (*G*/*G*_0_, where *G*_0_=2 *e* ^2^/*h*) of each junction varies as the STM tip is retracted from the surface (increasing distance, *z*) for a large number of traces (see the Supporting Information for details on the procedure and data from multiple experimental runs). All compounds give well‐defined plateaus, which become longer as the length of the molecule increases, indicating that connection occurs at the SMe groups (see Table 1 for experimental and calculated molecular lengths, *L*_exp_ and *L*_calc_, and Figure S9 for the plateau‐length distributions). The percentage of junctions giving a plateau for each compound is given in the caption to Figure 3 (and in Table S1). The measured conductances for the alkene and allene vary slightly with distance across the plateau (*z*). The molecular conductances plotted in Figure 3 g and listed in Table 1 are quoted from the histograms in Figure 3 f, where we have considered only points from the end of the distribution, greater than the median length determined from a Gaussian fit (see the Supporting Information for details). We had previously validated this method for a family of porphyrins.^17^ This analysis procedure ensures that the conductance values can be attributed to fully stretched single‐molecule junctions. It also results in good overlap between histograms (of the same compound) from periods of measurement giving different percentages of molecular junctions. We often observed an apparent increase in the molecular conductance when the percentage of molecular junctions exceeded about 40 %, which was attributed to the formation of junctions containing several wired molecules.^18^ However, if we only consider the data points at longer *z* values, the resulting histograms are more reproducible, indicating that the probability of multiple‐molecule junctions diminishes as the junctions are stretched (as discussed in Sections S3.8 and S3.9).

**Figure 3:**
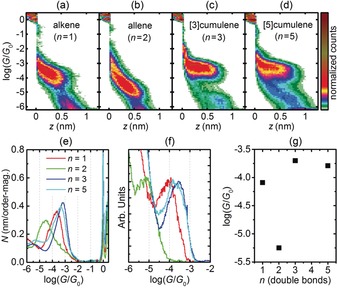
STM‐BJ results on molecules **1**–**3** and **5** measured with a bias of 0.2 V. a–d) 2D conductance histograms for the plateau‐containing traces. The number of traces in each (and percentage of the total) are as follows: *n*=1: 1309 (56 %), *n*=2: 2988 (58 %), *n*=3: 1139 (47 %), *n*=5: 3402 (34 %). e) 1D conductance histograms using all data points. f) 1D conductance histogram using only points from the end of the plateau distribution g) Molecular conductances from Gaussian fits to the data in (f); the experimental uncertainties in these values are smaller than the size of the black squares.

**Table 1:** Single‐molecule conductances, lengths, and HOMO–LUMO gaps for compounds **1**–**3** and **5**.

Compound	log(*G*/*G*_0_)^[a]^	*L*_exp_ [nm]^[b]^	*L*_calc_ [nm]^[c]^	*E*_g_(UV) [eV]^[d]^	*E*_g_(DFT) [eV]^[e]^
**1** (*n*=1)	−4.09	0.94 (1.32)	1.45	3.7	2.21
**2** (*n*=2)	−5.25	1.01 (1.34)	1.47	4.2	2.74
**3** (*n*=3)	−3.74	1.07 (1.46)	1.63	2.7	1.54
**5** (*n=*5)	−3.64	1.09 (1.64)	1.80	2.4	1.24

[a] Experimental conductance peak positions from data in Figure 3 f; the run‐to‐run variation in peak position is about 0.02, see Figure S19. [b] The lengths were calibrated by adding 0.4 nm to the peak position of a Gaussian fit to the total distribution of plateau lengths.2d, 17 Values in parentheses are derived from the 95th percentile. See Section 3.4 for the length distribution histograms. [c] Calculated using the Spartan quantum chemical package at the semi‐empirical level. We bound a gold atom to each sulfur and measured the Au–Au distance. [d] HOMO–LUMO gap calculated from the peak wavelength of the lowest‐energy absorption band in chloroform. [e] Calculated Kohn–Sham HOMO–LUMO gaps from DFT for isolated molecules in vacuum.

The [3] and [5]cumulenes have essentially the same conductance, which is slightly larger than that of the alkene (see Figure S19 for a comparison of multiple experimental runs showing high reproducibility). The allene has a conductance that is about 50 times smaller than those of the [3]‐ and [5]cumulenes, reflecting its twisted geometry. The conductances of all compounds show a weak voltage dependence, suggesting that the Fermi level (*E*_F_) lies far from any molecular levels, which is consistent with *E*_F_ sitting near the center of the HOMO–LUMO gap (see Section S3.5). Junctions of [5]cumulene were stable generally to ±1.2 V, whereas the shorter allene was stable only to ±0.5 V. At 1.2 V, the maximum current through a [5]cumulene molecule approaches microamperes (see Figure S10).

To gain further insight into the conductance trends in the family of cumulene molecular wires, and how their conductances change with length, transmission spectra *T*(*E*) were calculated by combining the DFT package SIESTA^19^ with the quantum transport code Gollum^20^ (for further details, see the Supporting Information). For the alkene, [3]cumulene, and [5]cumulene, the energetically preferred conformations have two terminal thioanisole rings that are not coplanar (see Section S2.2 for details), in agreement with crystallographic studies.^15^, ^21^ The conformations of relaxed molecules embedded in Au–Au junctions are shown in Figure 4 a. The frontier molecular orbitals (Figure S3) show that the HOMO of the alkene as well as the HOMO and LUMO of [3]cumulene and [5]cumulene form extended π‐conjugated transport paths through the whole molecules. In contrast, the molecular orbitals of the allene and [4]cumulene are formed from p_*z*_ and p_*x*_ orbitals and follow chiral paths through the molecules, leading to terminal thioanisole rings that are orthogonal to each other, and the absence of an extended π‐system in the HOMO and LUMO. As shown in Figure 4 b, these differences between the even‐ and odd‐numbered cumulenes are reflected in their transmission functions. Over a range of *E*_F_ within the HOMO–LUMO gap, indicated by the shaded region in Figure 4 b, the conductances of the allene and the [4]cumulene are expected to be lower than those of the other three molecules. More interestingly, the conductances of the [3]cumulene and the [5]cumulene are predicted to be about the same, despite the substantial change in length. This prediction is consistent with previous computational studies of charge transport through cumulene molecular wires.^4^, ^9^ The local density of states (LDOS) of the alkene and allene (Figure 4 c) reveals a clear transport path for the alkene (magenta surface), while there is no such LDOS path on the allene molecule. The alkene is predicted to have a slightly lower conductance than both the [3]‐ and [5]cumulenes because of the larger angle between the two thioanisole rings in the alkene, which is the result of steric interactions between the thioanisole and phenyl substituents.

**Figure 4:**
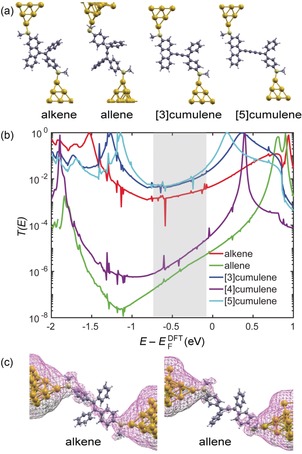
a) Calculated conformations for alkene, allene, [3]cumulene, and [5]cumulene attached to two gold leads, where the gray, white, and pale‐yellow balls represent carbon, hydrogen, and sulfur, respectively. The yellow balls at both ends represent gold leads. b) Transmission spectra. The shaded region indicates the range of Fermi energies within the HOMO–LUMO gap that contribute towards conduction at room temperature. c) The LDOS with magenta color in the energy window from −0.5 eV to 0 eV for the alkene and allene incorporated into two gold leads separately at the isosurface 0.00002.

The experimental conductances of molecules **1**, **2**, **3**, and **5** correlate well with their HOMO–LUMO gaps, as illustrated by the optical gaps, *E*_g_(UV), from the wavelength of the lowest‐energy UV/vis absorption band, and the Kohn–Sham gaps, *E*_g_(DFT), in Table 1.^22^ The lack of attenuation in the conductance for the series of compounds alkene, [3]cumulene, and [5]cumulene can be attributed to the decreasing HOMO–LUMO gaps, which compensate for the increasing length.^13^, ^23^ The lower molecular conductance of the allene is consistent with the large HOMO–LUMO gap, which leads to a higher barrier to electron transport. “Odd–even” conductance oscillations are anticipated from previous studies on metal atomic chains (e.g., Au, Pt, and Ir) as a function of the number of atoms in the chain.^24^ For example, Au and Na chains, with single conductance channels, show an odd–even effect that originates from quantum interference.^25^, ^26^ Odd–even oscillations have also been predicted for monoatomic carbon chains between carbon nanotubes.^27^ The odd–even effect that we observed here is probably dominated by the fact the 4‐thioanisole groups connecting the chains to the electrodes are only π‐conjugated in the odd [*n*]cumulenes (i.e., even number of carbon atoms). Another obvious difference between our measurements and the metal atomic chains is the fact that the chain and electrode atoms differ, producing an energy offset between the molecule and the electrodes.

In conclusion, we have revealed that the conductance of a series of cumulenes shows remarkably little dependence on the molecular length (*n*). This behavior is a consequence of the lack of strong BLA in these compounds, which results in a steep reduction in the HOMO–LUMO gap with increasing length.^6^, ^7^ In contrast, polyynes show strong BLA, resulting in an exponential attenuation of conductance with length (*β*≈0.2–0.3 Å^−1^ at low bias voltage).^28^ The [5]cumulene exhibits a high conductance of log(*G*/*G*_0_)=−3.7(±0.5) and shows stable junctions up to a bias of 1.2 V. The discovery that cumulenes exhibit length‐independent conductance suggests that they might be used to construct longer highly conductive molecular wires; however, this would require the development of effective strategies for controlling the reactivity of long cumulenes.^7^, ^29^

## Conflict of interest

The authors declare no conflict of interest.

## Supporting information

As a service to our authors and readers, this journal provides supporting information supplied by the authors. Such materials are peer reviewed and may be re‐organized for online delivery, but are not copy‐edited or typeset. Technical support issues arising from supporting information (other than missing files) should be addressed to the authors.

Supplementary
