# Supplementary material for: Unusual Length Dependence of the Conductance in Cumulene Molecular Wires
Source: Angew Chem Int Ed Engl. 2019 May 17;58(25):8378–82. doi: 10.1002/anie.201901228 (PMC6563095; doi:10.1002/anie.201901228)
Supplement: Supplementary file 1 — Supplementary [file ANIE-58-8378-s001.pdf]

Angewandte Chemie
Eine Zeitschrift der Gesellschaft Deutscher Chemiker

Supporting Information

Unusual Length Dependence of the Conductance in Cumulene Molecular Wires

Wenjun Xu[+], Edmund Leary[+], Songjun Hou[+], Sara Sangtarash, M. Teresa González, Gabino Rubio-Bollinger, Qingqing Wu, Hatef Sadeghi, Lara Tejerina, Kirsten E. Christensen, Nicolás Agraït, Simon J. Higgins, Colin J. Lambert,* Richard J. Nichols,* and Harry L. Anderson**

anie_201901228_sm_miscellaneous_information.pdf

Supporting Information

Table of Contents

	page
S1. Synthesis	S2
S1.1. General Synthetic Experimental Methods	S2
S1.2. Synthetic Schemes	S2
S1.3. Synthesis of Alkene **1**	S3
S1.4. Synthesis of Allene **2**	S3
S1.5. Synthesis of [3]Cumulene **3**	S4
S1.6. Synthesis of [5]Cumulene **5**	S6
S1.7. Single Crystal Data for Alkene **1**	S7
S1.8. UV-Vis Absorption Spectra	S8
S2. Theory	S9
S2.1. Computational Methods	S9
S2.2. Molecule Structures Used in Simulation	S10
S2.3. Calculations on Conformations with Terminal Anisole Rings Coplanar	S12
S2.4. The Effect of Rotating One of the Two Terminal Thioanisole Rings	S13
S2.5. Effects of Rotating the Phenyl Rings and *E/Z* Stereochemistry	S14
S3. STM Break-Junction Measurements	S15
S3.1. Sample Preparation for Single-Molecule Experiments	S15
S3.2. Single-Molecule Conductivity Studies	S15
S3.3. 2D Histograms	S16
S3.4. Plateau Length Distributions	S17
S3.5. Voltage Dependence of Molecular Conductance	S18
S3.6. Current through [5]Cumulene	S19
S3.7. 4,4'-Bis(methylthio)biphenyl	S19
S3.8. High *versus* Low Percentages of Molecular Junctions	S20
S3.9. Measuring the Conductance at the End of the Plateau Length Distribution	S21
S4. NMR Spectra of New Compounds	S24
S5. References	S34

Section S1.1. General Synthetic Experimental Methods

All manipulations of air- or water-sensitive compounds were performed using standard high-vacuum techniques. Commercially available reagents were used without further purification. Dry THF for reactions was purified by the solvent drying system MBraun MB-SPS-5-BenchTop under nitrogen atmosphere (H_2O content < 20 ppm as determined by Karl-Fischer titration). Unless specified otherwise, all other solvents were used as commercially supplied. Column chromatography was carried out using SiO_2 60 Å as stationary phase. Petroleum ether (PE) 40−60 °C was used unless specified otherwise. 4-Ethynylthioanisole was prepared as reported previously.[1]

^{1}H/^{13}C NMR spectra were recorded at 298 K using a Bruker AVIIIHD 400 nanobay or Bruker AVII 500 with ^{13}C cryoprobe. NMR spectra are reported in ppm; coupling constants (J) are reported in Hertz, to the nearest 0.1 Hz. Chemical shifts δ are calibrated by the residual solvent signals ($CDCl_3$: δ_H = 7.26 ppm, δ_C = 77.0 ppm; CD_2Cl_2: δ_H = 5.32 ppm, δ_C = 53.8 ppm; d_6-DMSO: δ_H = 2.50 ppm, δ_C = 39.5 ppm). UV-vis absorbance measurements were recorded in solution at 298 K using a Perkin-Elmer Lambda 20 spectrophotometer with quartz 1 cm cuvettes. Molar absorption coefficients are reported in L mol^{-1} cm^{-1}.

S1.2. Synthetic Schemes

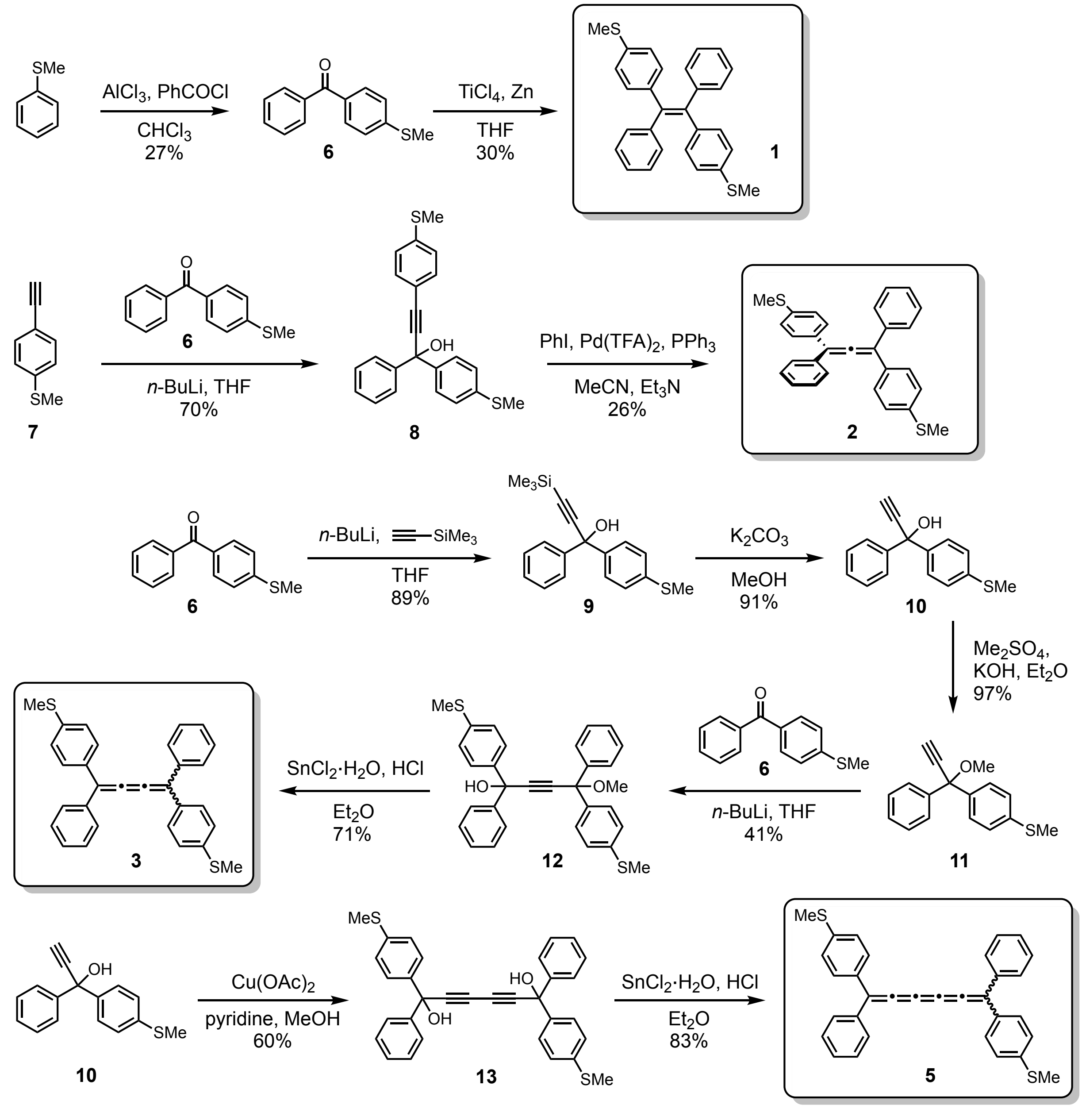

S1.3 Synthesis of Alkene 1

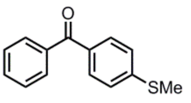

Synthesis of 4-methylthio-benzophenone (6). This compound was prepared using a modification of a published procedure.[2] Benzoyl chloride (7.0 mL, 60 mmol) was added to a solution of $AlCl_3$ (6.5 g, 48 mmol) in $CHCl_3$ (100 mL) with stirring at 0 °C. After the solid was completely dissolved, a solution of thioanisole (5.0 mL, 40 mmol) in $CHCl_3$ (20 mL) was added dropwise. The cooling bath was removed and the mixture was stirred at room temperature for 5 h. The mixture was poured into ice water with stirring and acidified with 2 M HCl until the solid dissolved. The organic layer was separated and washed with H_2O, saturated aqueous $NaHCO_3$, brine, and dried over Na_2SO_4. The solvent was removed under vacuum and recrystallization form ethanol (10 mL) yielded 4-methylthio-benzophenone **6** (2.44 g, 27%) as a white solid.

^{1}H NMR (400 MHz, $CDCl_3$, 298 K): δ_H 7.78−7.73 (m, 4H; H_{Ar}), 7.59−7.55 (m, 1H; H_{Ar}), 7.49−7.45 (m, 2H; H_{Ar}), 7.29 (d, J = 8.5 Hz, 2H; H_{Ar}), 2.53 (s, 3H; -SCH_3) ppm.

^{13}C NMR (100 MHz, $CDCl_3$, 298 K): δ_C 195.9, 145.4, 137.9, 133.7, 132.3, 130.7, 129.9, 128.4, 124.9, 14.9 ppm.

HR MS (ESI+): *m/z* 229.0682 ($[M+H]^+$ 100%, $C_{14}H_{13}O^{32}S^+$ requires 229.0682).

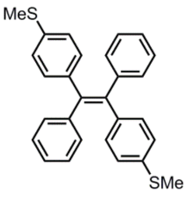

Synthesis of *E*-1,2-bis(4-(methylthio)phenyl)-1,2-diphenylethene (1). This compound was prepared using a procedure developed by Fang et al.[3] $TiCl_4$ (0.60 mL, 5 mmol) was added to dry THF (10 mL) under argon at 0 °C, then Zn dust (0.70 g, 10 mmol) was added to the mixture. The suspension was heated to reflux for 2 h. 4-Methylthio-benzophenone **6** (0.46 g, 2.0 mmol) was added to the suspension and kept at reflux under argon for 12 h. The reaction mixture was cooled to room temperature and treated with aqueous K_2CO_3 (10%). The organic layer was separated, and the aqueous suspension was extracted with CH_2Cl_2. The organic phase was dried with anhydrous Na_2SO_4, and the solvent was removed under vacuum. The crude product was purified by column chromatography (PE/EtOAc 7:1) and recrystallization from CH_2Cl_2/PE yielded alkene **1** as white needle crystals (0.13 g, 30%).

^{1}H NMR (400 MHz, $CDCl_3$, 298 K): δ_H 7.14−7.10 (m, 6H; H_{Ar}), 7.05−7.02 (m, 4H; H_{Ar}), 6.96 (d, J = 8.6 Hz, 4H; H_{Ar}), 6.91 (d, J = 8.7 Hz, 4H; H_{Ar}), 2.41 (s, 6H; -SCH_3) ppm.

^{13}C NMR (100 MHz, $CDCl_3$, 298 K): δ_C 143.8, 140.6, 140.3, 136.5, 131.9, 131.5, 128.0, 126.7, 125.6, 15.6 ppm.

HR MS (EI+): *m/z* 424.1311 ($[M]^+$ 100%, $C_{28}H_{24}{}^{32}S_2{}^+$ requires 424.1314).

UV-vis ($CHCl_3$): λ / nm (ε / M^{-1} cm^{-1}): 336 (1.9 × 10^4), 271 (2.5 × 10^4).

Melting point: 209−210 °C.

S1.4. Synthesis of Allene 2

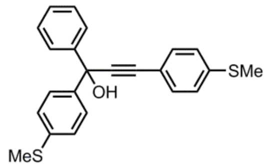

Synthesis of 1,3-bis(4-(methylthio)phenyl)-1-phenylprop-2-yn-1-ol (8).[4] 4-Ethynylthioanisole **7** (0.33 g, 2.2 mmol) was dissolved in dry THF (8 mL) and cooled to −78 °C. *n*-BuLi (1.6 M in hexane, 1.5 mL, 2.4 mmol) was added dropwise under argon. The solution was stirred for 1 h at −78 °C, then 4-methylthio-benzophenone **6** (0.38 g, 1.7 mmol) was added as a solution in dry THF (8 mL). The cooling bath was removed and the solution was stirred at room temperature overnight. The reaction mixture was treated with saturated aqueous NH_4Cl (15 mL). The aqueous layer was extracted with CH_2Cl_2 and the organic phase was washed with water. Evaporation and column chromatography (PE/EtOAc 9:1) yielded 1,3-bis(4-(methylthio)phenyl)-1-phenylprop-2-yn-1-ol **8** (0.44 g, 70%) as a viscous yellow oil.

^{1}H NMR (400 MHz, $CDCl_3$, 298 K): δ_H 7.71–7.68 (m, 2H; H_{Ar}), 7.61 (d, J = 8.7 Hz, 2H; H_{Ar}), 7.42 (d, J = 8.7 Hz, 2H; H_{Ar}), 7.39–7.35 (m, 2H; H_{Ar}), 7.32–7.28 (m, 1H; H_{Ar}), 7.23 (d, J = 8.7 Hz, 2H; H_{Ar}), 7.19 (d, J = 8.6 Hz, 2H; H_{Ar}), 3.23 (s, 1H; -OH), 2.47 (s, 3H; -SCH_3), 2.46 (s, 3H; -SCH_3) ppm.

^{13}C NMR (100 MHz, $CDCl_3$, 298 K): δ_C 144.9, 142.0, 139.9, 138.0, 132.0, 128.3, 127.8, 126.6, 126.2, 126.0, 125.7, 118.5, 91.7, 87.0, 77.5, 77.2, 76.8, 74.5, 15.7, 15.3 ppm.

HR MS (ESI+): *m/z* 377.1029 ($[M+H]^+$ 100%, $C_{23}H_{21}O^{32}S_2^+$ requires 377.1028).

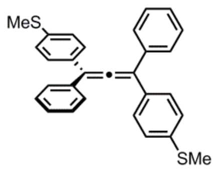

Synthesis of 1,3-bis(4-(methylthio)phenyl)-1,3-diphenylpropa-1,2-diene (2).[5] Acetonitrile (16 mL) and triethylamine (0.4 mL) were deoxygenated and saturated with argon, then added to a mixture of 1,3-bis(4-(methylthio)phenyl)-1-phenylprop-2-yn-1-ol **8** (200 mg, 0.5 mmol), iodobenzene (240 mg, 1.2 mmol), $Pd(TFA)_2$ (10 mg, 0.03 mmol) and triphenylphosphine (20 mg, 0.08 mmol) under argon. The solution was stirred at 80 °C for 24 h. After cooling to room temperature, the solvent was removed under vacuum, and the residue was filtered through a SiO_2 plug. Column chromatography (PE/EtOAc 95:5) gave 1,3-bis(4-(methylthio)phenyl)-1,3-diphenylpropa-1,2-diene **2** (60 mg, 26%) as a yellow oil.

^{1}H NMR (400 MHz, CD_2Cl_2, 298 K): δ_H 7.42–7.29 (m, 14H; H_{Ar}), 7.23 (d, J = 8.7 Hz, 4H; H_{Ar}), 2.49 (s, 6H; -SCH_3) ppm.

^{13}C NMR (125 MHz, CD_2Cl_2, 298 K): δ_C 208.7, 138.6, 136.5, 133.2, 129.04, 128.96, 128.7, 128.0, 126.8, 112.8, 15.9 ppm.

HR MS (APCI+): *m/z* 437.1395 ($[M+H]^+$ 100%, $C_{29}H_{25}{}^{32}S_2^+$ requires 437.1392).

UV-vis ($CHCl_3$): λ / nm (ε / M^{-1} cm^{-1}): 297 (3.0×10^4), 265 (2.9×10^4).

S1.5. Synthesis of [3]Cumulene

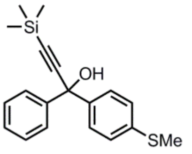

Synthesis of 1-(4-(methylthio)phenyl)-1-phenyl-3-(trimethylsilyl)prop-2-yn-1-ol (9).[4] *n*-BuLi (1.6 M in hexane, 4.7 mL, 7.5 mmol) was added dropwise to a solution of ethynyltrimethylsilane (1.1 mL, 7.5 mmol) in dry THF (12.5 mL) at −78 °C under argon. After stirring for 0.5 h at −78 °C, 4-methylthio-benzophenone **6** (0.86 g, 3.8 mmol) was added as a solution in dry THF (5 mL). The mixture was stirred at room temperature for 17 h, then treated with saturated aqueous NH_4Cl (19 mL) and extracted with diethyl ether. Solvent was removed under vacuum and column chromatography (PE/EtOAc 9:1) gave 1-(4-(methylthio)phenyl)-1-phenyl-3-(trimethylsilyl)prop-2-yn-1-ol **9** (1.10 g, 89%) as a viscous yellow liquid.

^{1}H NMR (400 MHz, $CDCl_3$, 298 K): δ_H 7.64–7.61 (m, 2H; H_{Ar}), 7.54 (d, J = 8.7 Hz, 2H; H_{Ar}), 7.38–7.33 (m, 2H; H_{Ar}), 7.31–7.27 (m, 1H; H_{Ar}), 7.23 (d, J = 8.6 Hz, 2H; H_{Ar}), 2.82 (s, 1H; -C≡CH), 2.49 (s, 3H; -SCH_3), 0.27 (s, 9H; -Si(CH_3)$_3$) ppm.

^{13}C NMR (100 MHz, $CDCl_3$, 298 K): δ_C 144.8, 141.9, 138.1, 128.4, 127.8, 126.6, 126.3, 126.1, 107.7, 92.2, 74.5, 15.8, 0.0 ppm.

HR MS (ESI+): *m/z* 349.1053 ($[M+Na]^+$ 100%, $C_{19}H_{22}O^{23}Na^{32}S^{28}Si^+$ requires 349.1053).

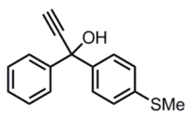

Synthesis of 1-(4-(methylthio)phenyl)-1-phenylprop-2-yn-1-ol (10). Potassium carbonate (3.33 g, 24.1 mmol) was added to a solution of 1-(4-(methylthio)phenyl)-1-phenyl-3-(trimethylsilyl)prop-2-yn-1-ol **9** (1.16 g, 3.6 mmol) in methanol (13 mL). After stirring for 2 h, the solvent was removed under vacuum and residue was filtered through a plug of SiO_2. Evaporation gave 1-(4-(methylthio)phenyl)-1-phenyl-3-(trimethylsilyl)prop-2-yn-1-ol **10** (0.82 g, 91%) as a pale yellow liquid.

^{1}H NMR (400 MHz, $CDCl_3$, 298 K): δ_H 7.59–7.56 (m, 2H; H_{Ar}), 7.49 (d, J = 8.6 Hz, 2H; H_{Ar}), 7.34–7.30 (m, 2H; H_{Ar}), 7.28–7.24 (m, 1H; H_{Ar}), 7.19 (d, J = 8.6 Hz, 2H; H_{Ar}), 2.86 (s, 1H; -C≡CH), 2.80 (bs, 1H; -OH), 2.44 (s, 3H; -SCH_3) ppm.

^{13}C NMR (100 MHz, $CDCl_3$, 298 K): δ_C 144.4, 141.4, 138.4, 128.5, 128.0, 126.6, 126.4, 126.0, 86.3, 75.7, 74.1, 15.8 ppm.

HR MS (ESI+): *m/z* 255.0839 ($[M+H]^+$ 100%, $C_{16}H_{15}O^{32}S^+$ requires 255.0838).

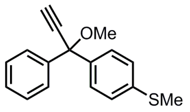

Synthesis of (4-(1-methoxy-1-phenylprop-2-yn-1-yl)phenyl)(methyl)sulfane (11).[4] Compound **9** (0.30 g, 1.2 mmol) was dissolved in diethyl ether (12 mL) and KOH (0.27 g, 4.8 mmol) was added. To this suspension, dimethyl sulfate (0.19 mL, 2.0 mmol) was added slowly. After stirring overnight, water (20 mL) was added and the aqueous phase was extracted with diethyl ether, then the solvent was removed under vacuum. Column chromatography (PE/EtOAc 95:5) gave (4-(1-methoxy-1-phenylprop-2-yn-1-yl)phenyl)(methyl)sulfane **11** (0.31 g, 97%) as a pale yellow oil.

^{1}H NMR (400 MHz, $CDCl_3$, 298 K): δ_H 7.56–7.53 (m, 2H; H_{Ar}), 7.47 (d, J = 8.6 Hz, 2H; H_{Ar}), 7.36–7.31 (m, 2H; H_{Ar}), 7.29–7.25 (m, 1H; H_{Ar}), 7.21 (d, J = 8.6 Hz, 2H; H_{Ar}), 3.36 (s, 3H; -OCH_3), 2.90 (s, 1H; -C≡CH), 2.47 (s, 3H; -SCH_3) ppm.

^{13}C NMR (100 MHz, $CDCl_3$, 298 K): δ_C 142.9, 140.0, 138.2, 128.3, 127.9, 127.3, 126.7, 126.3, 83.0, 80.5, 77.8, 52.6, 15.8 ppm.

HR MS (APCI+): *m/z* 269.0996 ($[M+H]^+$ 100%, $C_{17}H_{17}O^{32}S^+$ requires 269.0995).

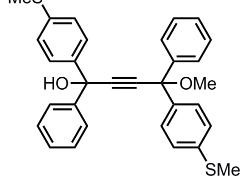

Synthesis of 4-methoxy-1,4-bis(4-(methylthio)phenyl)-1,4-diphenylbut-2-yn-1-ol (12).[4] Compound **11** (0.15 g, 0.6 mmol) was dissolved in dry THF (3 mL) and cooled to −78 °C. *n*-BuLi (1.6 M in hexane, 0.4 mL, 0.6 mmol) was added dropwise under argon. The solution was stirred for 1 h at the same temperature, then 4-methylthio-benzophenone **6** (0.10 g, 0.4 mmol) was added as a solution in dry THF (4 mL). The cooling bath was removed and the solution was stirred at room temperature overnight. The reaction mixture was treated with saturated aqueous NH_4Cl (15 mL). Aqueous layer was extracted with diethyl ether and the organic phase was washed with H_2O and brine. Solvent was removed in vacuum and column chromatography (use PE/EtOAc 95:5 → PE/EtOAc 9:1 to removed impurities then CH_2Cl_2) gave 4-methoxy-1,4-bis(4-(methylthio)phenyl)-1,4-diphenylbut-2-yn-1-ol **12** (90 mg, 41%) as a pale yellow oil.

^{1}H NMR (400 MHz, d_6-DMSO, 298 K): δ_H 7.56–7.54 (m, 2H; H_{Ar}), 7.53–7.50 (m, 2H; H_{Ar}), 7.49–7.43 (m, 4H; H_{Ar}), 7.37–7.31 (m, 4H; H_{Ar}), 7.29–7.21 (m, 6H; H_{Ar}), 7.02 (s, 1H; -OH), 3.30 (s, 3H; -OCH_3), 2.45 (s, 3H; -SCH_3), 2.44 (s, 3H; -SCH_3) ppm.

^{13}C NMR (100 MHz, d_6-DMSO, 298 K): δ_C 146.0, 143.2, 142.8, 139.7, 137.8, 137.2, 128.3, 128.1, 127.7, 127.3, 126.7, 126.1, 126.0, 125.6, 125.5, 93.2, 84.6, 80.2, 72.8, 52.2, 14.6, 14.5 ppm.

HR MS (ESI+): *m/z* 519.1422 ($[M+Na]^+$ 100%, $C_{31}H_{28}O_2{}^{23}Na^{32}S_2{}^+$ requires 519.1423).

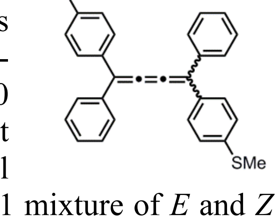

Synthesis of 1,4-bis(4-(methylthio)phenyl)-1,4-diphenylbuta-1,2,3-triene (3).[4] Hydrogen chloride (1.0 M solution in Et_2O, 1.5 mL, 1.5 mmol) was added *via* syringe to a solution of 4-methoxy-1,4-bis(4-(methylthio)phenyl)-1,4-diphenylbut-2-yn-1-ol **12** (70 mg, 0.14 mmol) and anhydrous $SnCl_2$ (70 mg, 0.37 mmol) in anhydrous diethyl ether (6 mL). After stirring for 3 h at room temperature, the mixture was filtered. The solid was washed with diethyl ether and water to give the [3]cumulene **3** (45 mg, 71%) as a yellow solid (1:1 mixture of *E* and *Z* isomers).

^{1}H NMR (500 MHz, CD_2Cl_2, 298 K): δ_H 7.57–7.53 (m, 4H; H_{Ar}), 7.50–7.46 (m, 4H; H_{Ar}), 7.43–7.33 (m, 6H; H_{Ar}), 7.28–7.24 (m, 4H; H_{Ar}), 2.53 (s, 3H; -SCH_3), 2.52 (s, 3H; -SCH_3) ppm.

^{13}C NMR (125 MHz, CD_2Cl_2, 298 K): δ_C 150.73, 139.43, 139.41, 139.00, 138.93, 135.73, 135.67, 129.96, 129.92, 129.70, 129.67, 128.87, 128.85, 128.42, 128.41, 126.26, 126.24, 122.17, 15.63, 15.62 ppm (two signals overlapped or not observed).

HR MS (EI+): *m/z* 448.1308 ($[M]^+$ 100%, $C_{30}H_{24}{}^{32}S_2{}^+$ requires 448.1314).

UV-vis ($CHCl_3$): λ / nm (ε / M^{-1} cm^{-1}): 453 (4.4×10^4), 301 (2.9×10^4).

Melting point: 252 °C.

S1.6. Synthesis of [5]Cumulene

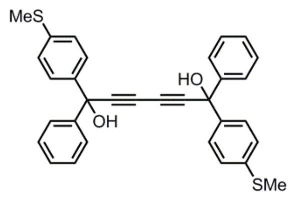

Synthesis of 1,6-bis(4-(methylthio)phenyl)-1,6-diphenylhexa-2,4-diyne-1,6-diol (13).[6] To a stirred solution of $Cu(OAc)_2$ (0.35 g, 1.6 mmol) in methanol/pyridine (1:1, 2.6 mL) was added a solution of **10** (0.20 g 0.8 mmol) in a mixture of methanol/pyridine (1:1, 2.6 mL) and the solution was stirred at 60 °C for 17 h. After cooling to room temperature, the solvent was removed under vacuum and extracted with diethyl ether. The organic phase was washed with 1 M HCl and brine, and then dried over Na_2SO_4. The organic phase was filtered through a plug of SiO_2 and solvent was removed under vacuum. A pale yellow oil was obtained and recrystallization from CH_2Cl_2/PE yielded the product **13** as a light brown solid (0.12 g, 60%).

^{1}H NMR (400 MHz, d_6-DMSO, 298 K): δ_H 7.49–7.46 (m, 4H, H_{Ar}), 7.40 (d, J = 8.4 Hz, 4H; H_{Ar}), 7.36–7.32 (m, 4H; H_{Ar}), 7.28–7.26 (m, 2H; H_{Ar}), 7.23 (d, J = 8.5 Hz, 4H; H_{Ar}), 7.13 (s, 2H; -OH), 2.44 (s, 6H; -SCH_3) ppm.

^{13}C NMR (100 MHz, d_6-DMSO, 298 K): δ_C 145.0, 141.8, 137.5, 128.2, 127.5, 126.2, 125.6, 125.6, 83.9, 73.0, 69.4, 14.6 ppm.

HR MS (APCI–): *m/z* 505.1300 ($[M–H]^-$ 100%, $C_{32}H_{25}O_2{}^{32}S_2{}^-$ requires 505.1301).

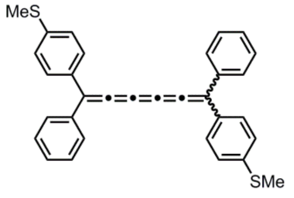

Synthesis of 1,6-bis(4-(methylthio)phenyl)-1,6-diphenylhexa-1,2,3,4,5-pentaene (5).[4] Hydrogen chloride (solution in Et_2O, 1.0 M, 1.5 mL, 1.5 mmol) was added to a solution of 1,6-bis(4-(methylthio)phenyl)-1,6-diphenylhexa-2,4-diyne-1,6-diol **13** (50 mg, 0.10 mmol) and anhydrous $SnCl_2$ (50 mg, 0.26 mmol) in diethyl ether (6 mL). After stirring for 8 h at room temperature, the mixture was filtered. The solid was washed with diethyl ether and water to give the [5]cumulene **5** (39 mg, 83%) as a red solid (1:1 mixture of *E* and *Z* isomers).

^{1}H NMR (500 MHz, CD_2Cl_2, 298 K): δ_H 7.57–7.55 (m, 4H; H_{Ar}), 7.51–7.49 (m, 4H; H_{Ar}), 7.44–7.35 (m, 6H; H_{Ar}), 7.27–7.25 (m, 4H; H_{Ar}), 2.53 (s, 3H; -SCH_3), 2.52 (s, 3H; -SCH_3) ppm.

^{13}C NMR (125 MHz, CD_2Cl_2, 298 K): δ_C 147.4, 140.1, 137.9, 134.6, 129.4, 129.3, 128.6, 128.6, 125.8, 123.8, 15.1 ppm.

HR MS (EI+): *m/z* 472.1313 ($[M]^+$ 100%, $C_{32}H_{24}{}^{32}S_2{}^+$ requires 472.1314).

UV-vis ($CHCl_3$): λ / nm (ε / M^{-1} cm^{-1}): 521 (7.0×10^4), 375 (1.6×10^4), 258 (4.6×10^4).

Melting point: >370 °C (dec.).

S1.7. Single Crystal data for 1,2-bis(4-(methylthio)phenyl)-1,2-diphenylethene (1).

Low temperature single crystal X-ray diffraction data were collected for compound **1** using a (Rigaku) Oxford Diffraction SuperNova diffractometer.[7] Raw frame data were reduced using CrysAlisPro and the structure was solved using 'Superflip'[8] before refinement with CRYSTALS[9] as per the CIF. Crystallographic data have been deposited with the Cambridge Crystallographic Data Centre (CCDC 1893619) and can be obtained *via* www.ccdc.cam.ac.uk/data_request/cif.

Table 1. Crystal data and structure refinement for (1).

Identification code	7097	
Empirical formula	C28 H24 S2	
Formula weight	424.63	
Temperature	150 K	
Wavelength	1.54184 Å	
Crystal system	Monoclinic	
Space group	P 2_1/c	
Unit cell dimensions	*a* = 5.5302(2) Å	α = 90°.
	b = 16.9720(4) Å	β = 90.056(3)°.
	c = 23.8523(6) Å	γ = 90°.
Volume	2238.74(11) $Å^3$	
Z	4	
Density (calculated)	1.260 Mg/m^3	
Absorption coefficient	2.229 mm^{-1}	
F(000)	896	
Crystal size	0.39 x 0.05 x 0.04 mm^3	
Theta range for data collection	4.531 to 76.356°.	
Index ranges	$-6 \le h \le 6$, $-20 \le k \le 21$, $-29 \le l \le 29$	
Reflections collected	23629	
Independent reflections	4633 [*R*(int) = 0.045]	
Completeness to theta = 74.829°	99.7 %	
Absorption correction	Semi-empirical from equivalents	
Max. and min. transmission	0.91 and 0.76	
Refinement method	Full-matrix least-squares on F^2	
Data / restraints / parameters	4633 / 0 / 272	
Goodness-of-fit on F^2	0.9991	
Final R indices [$I > 2\text{sigma}(I)$]	*R*1 = 0.0384, *wR*2 = 0.0979	
R indices (all data)	*R*1 = 0.0431, *wR*2 = 0.1048	
Largest diff. peak and hole	0.18 and -0.37 e.$Å^{-3}$	

ORTEP drawing of **1** with thermal ellipsoids drawn at 50% probability:

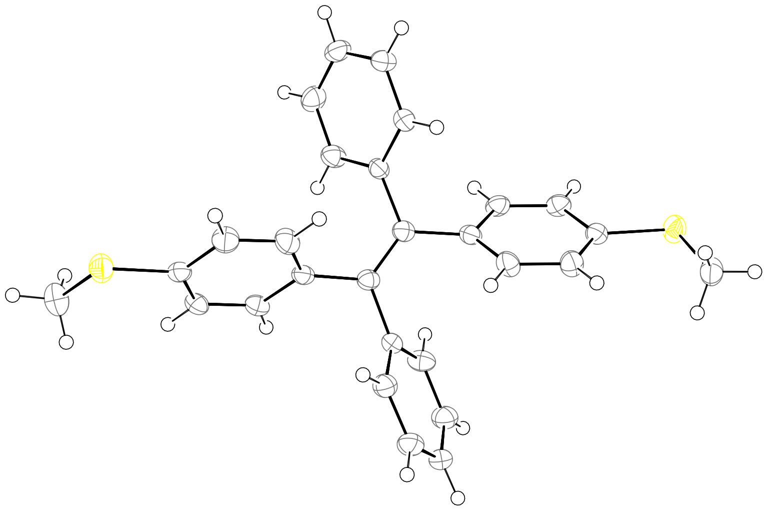

S1.8. UV-Visible Absorption Spectra

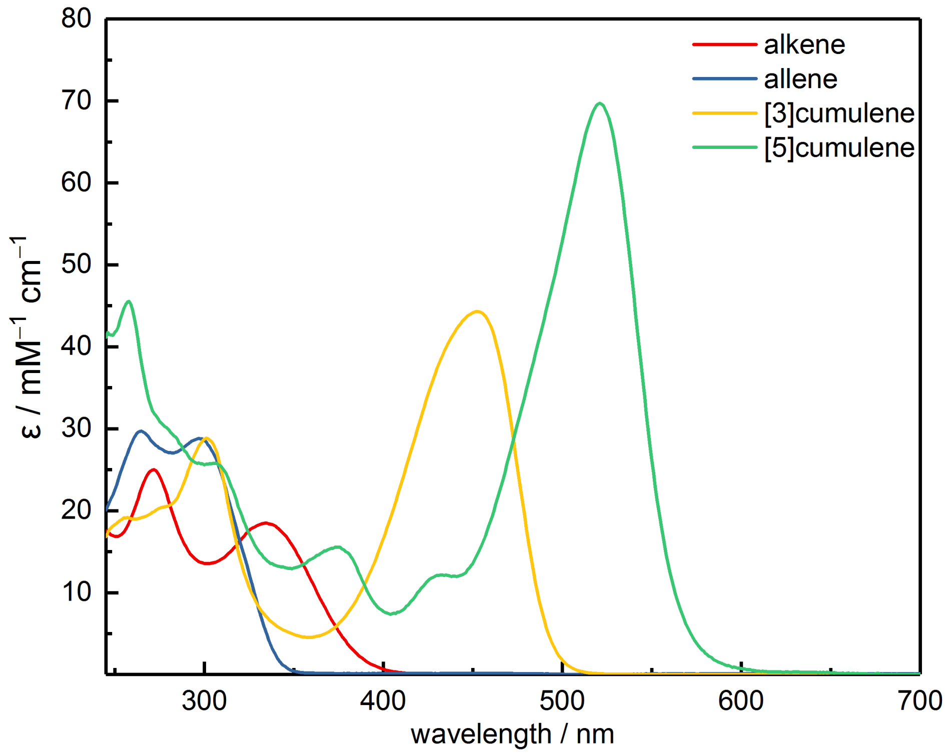

Figure S1. UV-vis absorption spectra of compounds **1**, **2**, **3** and **5** recorded in chloroform.

Section S2.1. Theory: Computational Methods

Geometrical optimizations were performed by using the DFT code SIESTA,[10] with a local density approximation GGA functional), double-ζ polarized basis, a cutoff energy of 150 Ry and a 0.05 eV/Å force tolerance. To compute their electrical conductance, the molecules were each placed between pyramidal gold electrodes. For each structure, the transmission coefficient $T(E)$ describing the propagation of electrons of energy E from the left to the right electrodes was calculated using Gollum[11] code, which combines the mean-field Hamiltonian and overlap matrices of the DFT code SIESTA with Landauer-based quantum transport theory transport theory, *via* the expression

$$T(E) = Tr[\Gamma_L(E) g(E) \Gamma_R(E) g^{\dagger}(E)] \qquad (1)$$

where $\Gamma_{L,R}(E) = i(\Sigma_{L,R}(E) - \Sigma_{L,R}{}^{\dagger}(E))/2$ is the imaginary part of the self-energies, $\Sigma_{L,R}(E)$, characterizing the contact between the molecule and left (L) and right (R) electrodes and g is the retarded Green's function of the molecule in the presence of the electrodes. $\Gamma_{L,R}$ determines the width of transmission resonances. The room-temperature conductance is extracted from the transmission spectrum and evaluated by the following formulae:

$$G = G_0 \int_{-\infty}^{+\infty} dE T(E) (-\frac{\partial f(E)}{\partial E}) \qquad (2)$$

where $G_0 = 2e^2/h$ is the conductance quantum; h is the Planck's constant; e is the charge of a proton; $f(E) = (1 + \exp(E - E_F/k_B T))^{-1}$ is the Fermi-Dirac probability distribution function, E_F is the Fermi energy.

As discussed in ref. [12], the predicted length-dependence of molecular conductance is sensitive to the relative position of E_F, and the value of this parameter may be incorrectly predicted by DFT. The calculations in that paper demonstrated that the length-dependence of the conductance, and other phenomena such as negative differential resistance, are sensitive to the Fermi energy. For this reason, it has become common to treat E_F as a fitting parameter when comparing theory with experiment. Wang et al measured a low beta value for a series of oligoynes,[13] which suggested that in those experiments, E_F was close to the LUMO, rather than being in the middle of the HOMO-LUMO gap, probably as a result of the high bias (0.6 V). In a later study, beta values ranging from 1.7 nm^{-1} to 3.2 nm^{-1} were measured for oligoynes with various terminal group using a much lower bias voltage of 0.035 V.[14] Again it was concluded that these differences arose from the different values of E_F, associated with the various anchor groups. In all of these studies, it was found that when E_F is located near the middle of the HOMO-LUMO gap, oligoynes possess a large positive beta value, in stark contrast with the cumulenes presented in here.

We carried out two further studies, beyond DFT, focusing on transport near the middle of the HOMO-LUMO gap. In ref. [15], we used GW many body theory to examine the HOMO-LUMO gap Δ of infinite oligoynes and from the asymptotic value of Δ = 2.16 eV, we predicted that the beta value of long oligoynes, with a E_F at the gap center, is 2.1 nm^{-1}. In a computationally-intensive Quantum Monte Carlo study,[16] we found the excitonic and quasiparticle gaps to be 3.30 and 3.4 eV, respectively, which may account for the largest value of beta measured in ref. [14]. It should be emphasized that this mid-gap behavior contrasts with the transport properties of cumulenes within the gap, where the transmission coefficient increases with length (Figure 4).

S2.2. Molecule Structures Used in Simulation

Since the cumulene wires have four phenyl rings, they are likely to be trapped in local energy minima during DFT relaxing process. Therefore, for alkene, [3]cumulene and [5]cumulene, two types of initial structures were used in which the two terminal thioanisole rings are initially planar or non-planar. The relaxed conformations as well as the corresponding energies are shown in Figure S2(a) and (b) respectively, which indicating that those in panel b are the more energy favorable conformations (indicated by red square in Figure S2(b)). Therefore the molecular structures in Figure S2(b) were used in our simulations. For [2]cumulene and [4]cumulene, whether or not we choose the two terminal thioanisole rings to be planar or non-planar, after relaxation, we obtain molecules with the two terminal thioanisole rings perpendicular to each other due to the odd number of carbon atoms with two π electrons (*e.g.* The middle carbon atom for allene, the middle three carbon atoms for [4]cumulene) (see the top of Figure S3 for the chosen allene conformations).

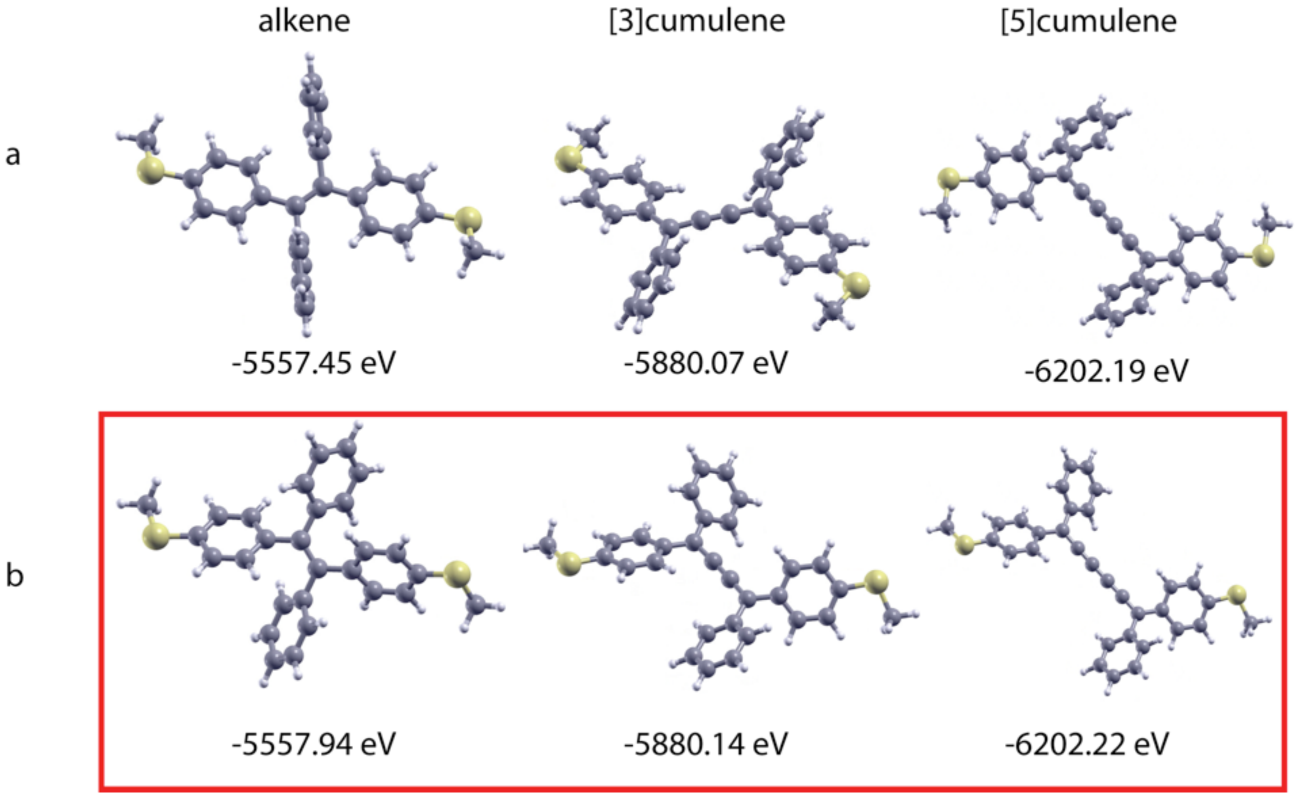

Figure S2. For alkene, [3]cumulene and [5]cumulene, the relaxed structures as well as the corresponding energy of two types of initial structures: (a) the two terminal thioanisole rings are coplanar; (b) the two terminal thioanisole rings are not coplanar. The conformations we chose for calculation in manuscript are those in panel (b) (in red square) because they are most stable.

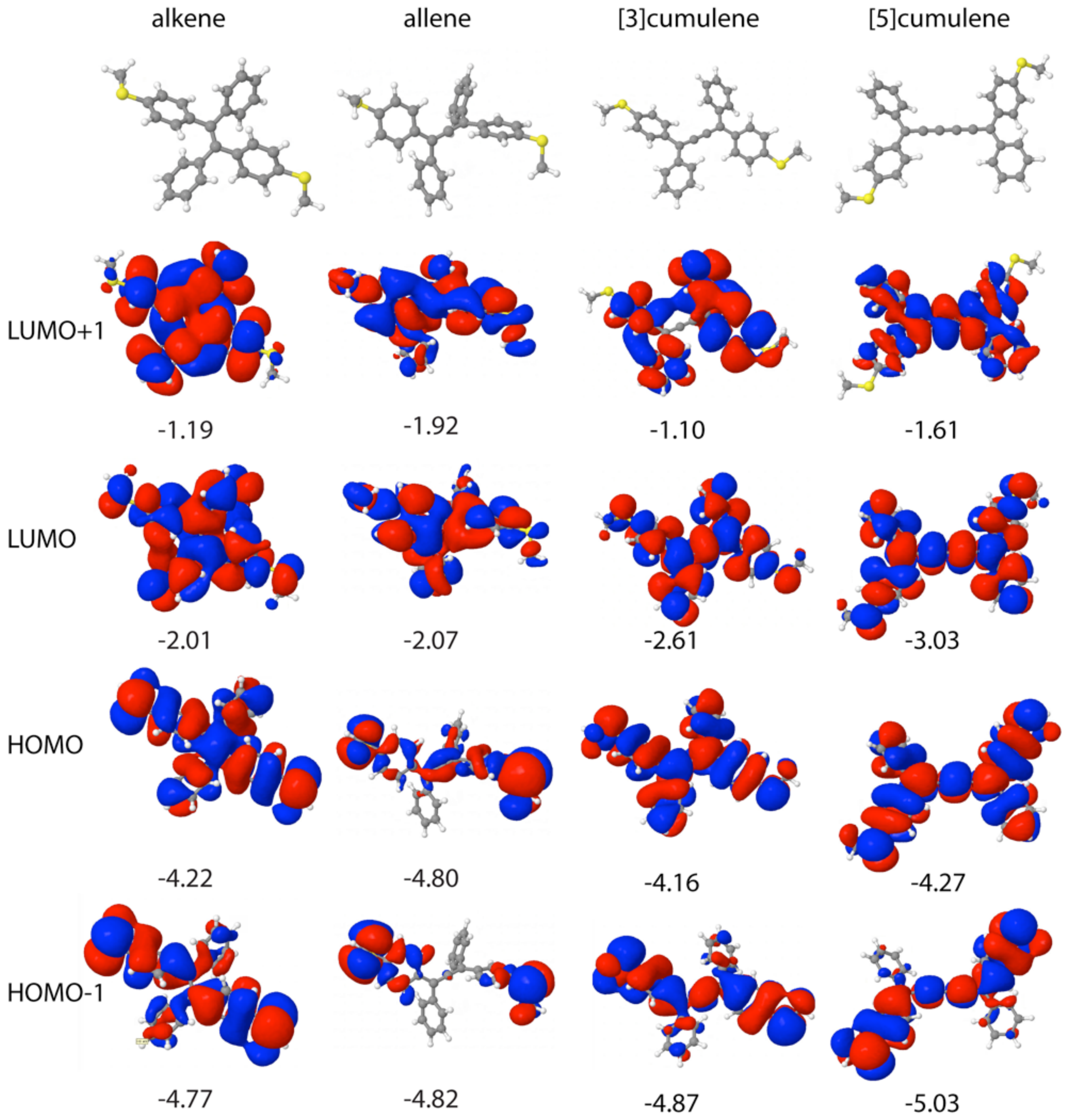

Figure S3. The frontier molecular orbitals HOMO-1, HOMO, LUMO and LUMO+1 of isolated molecules of alkene, allene, [3]cumulene and [5]cumulene as well as the corresponding energy eigenvalues (in eV).

S2.3. Calculations on Conformations with Terminal Anisole Rings Coplanar

In this section, the conformations in Figure S2(a) were used to compute the change in conductances with length. The results demonstrating that the trend predicted in manuscript (*i.e.* the conductance of [3]cumulene and [5]cumulene are similar, and both are slightly higher than that of alkene) is again obtained using these different conformations.

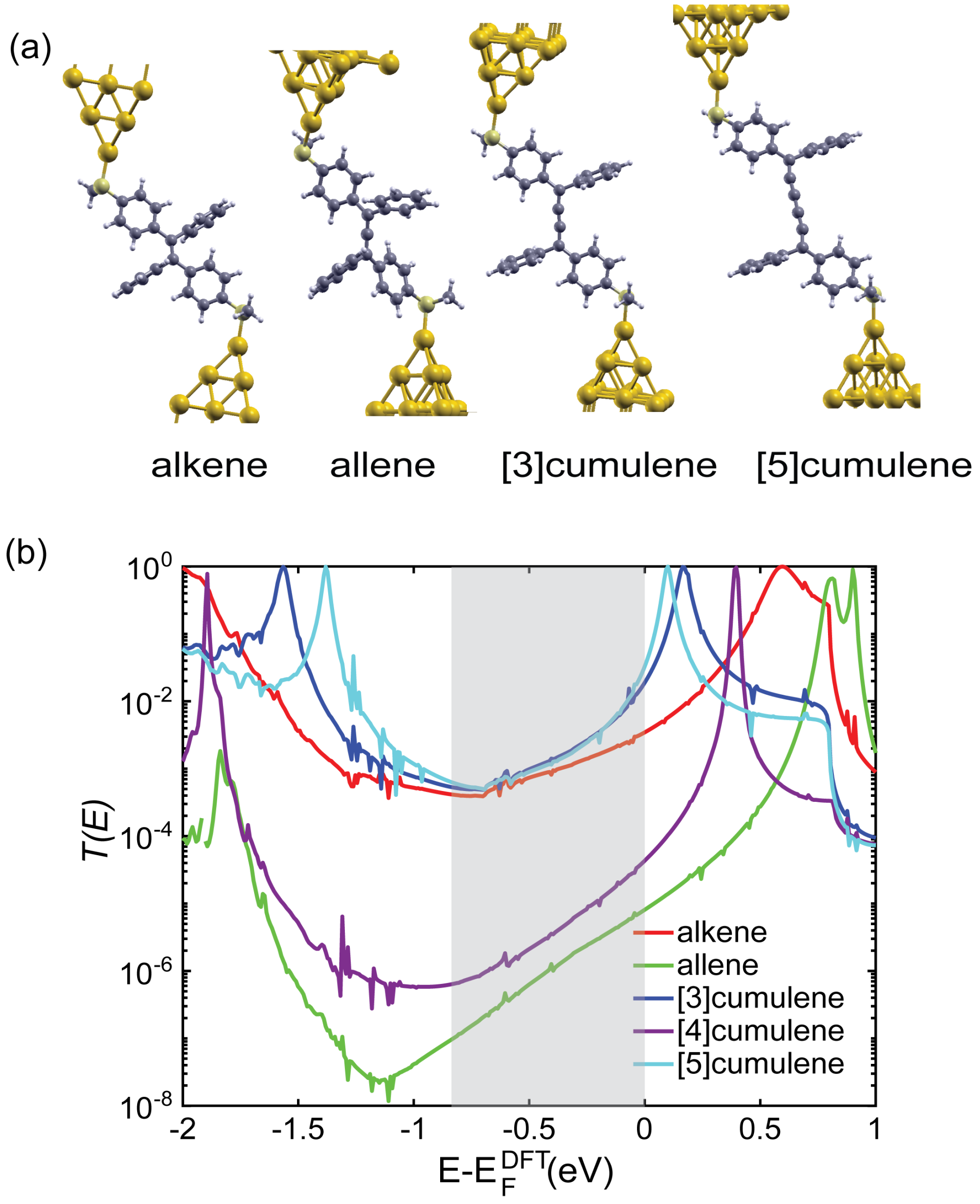

Figure S4. (a) Calculated conformations for alkene, allene, [3]cumulene, [5]cumulene attached to two gold leads with the thioanisole rings coplanar (grey, white and pale yellow balls represent carbon, hydrogen and sulfur, respectively). The yellow balls at both ends represent gold leads. (b) Transmission spectra. The shaded region indicates the range of Fermi energies within the HOMO-LUMO gap that contribute towards conduction at room temperature. For alkene, [3]cumulene and [5]cumulene, the conformations in Figure S2(a) are used, which are essentially the same as those in Figure S4(a).

S2.4 The Effect of Rotating One of the Two Terminal Thioanisole Rings

Here we explore the change in the transmission coefficient due to rotation of one phenyl ring (indicated by the red arrow on the top of Figure S5) by 20°, 40° and 60°. The corresponding transmission functions indicate that the above trend (the conductance of [3]cumulene and [5]cumulene are similar, and both are slightly higher than that of alkene) does not change.

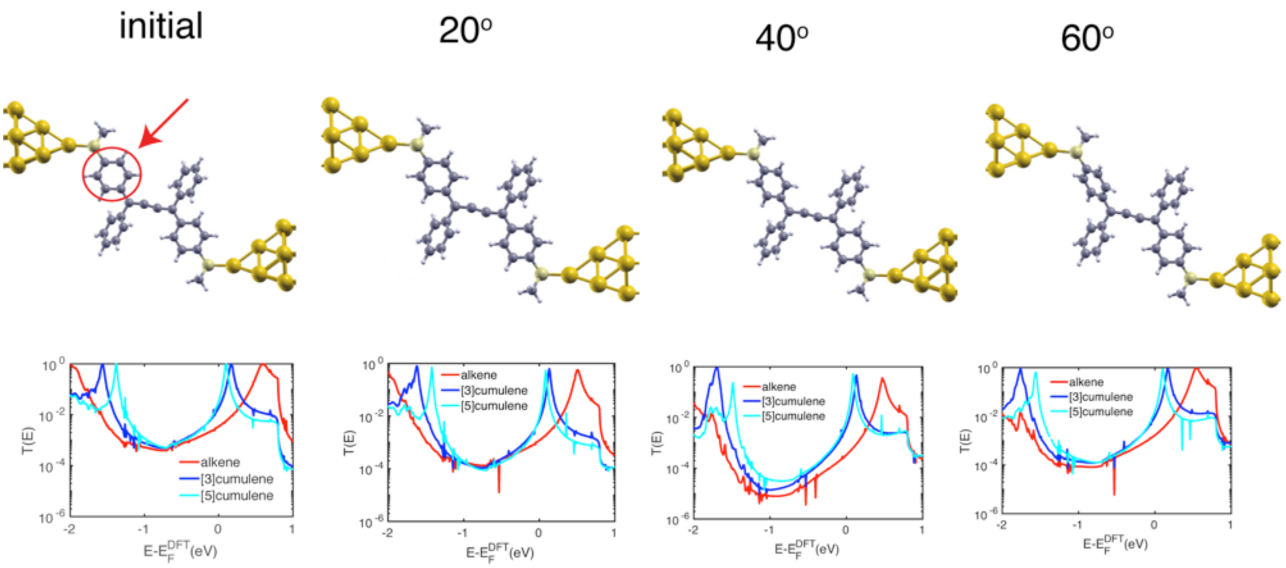

Figure S5. Transmission function *versus* the orientation of one of the two terminal phenyl rings relative to the other terminated phenyl ring for alkene, [3]cumulene and [5]cumulene. The red arrow indicates the phenyl ring which is rotated by 20°, 40° and 60°.

S2.5 The Effect of Rotating the Two Side Phenyl Rings and *E/Z* Stereochemistry

To clarify the effect of the two internal, pendant phenyl rings, taking [5]cumulene as an example, Figure S3 shows the effect of artificially rotating the rings by 25°, 50°, 75° which indicate the conformation of these rings has only a small effect on the transmission coefficient within the gap.

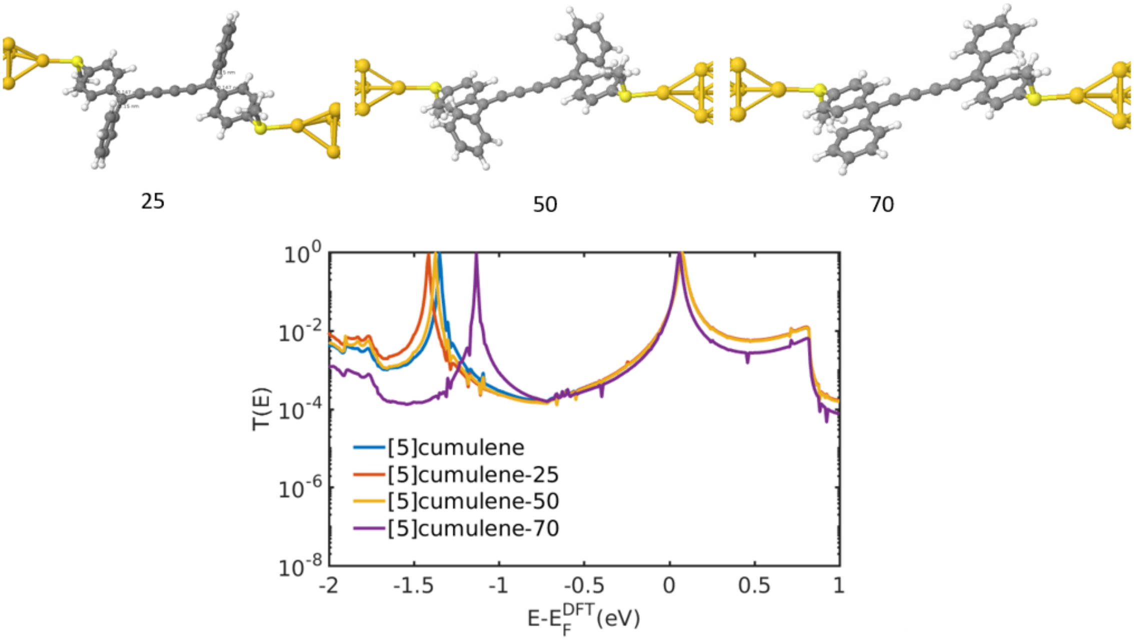

Figure S6. Transmission function *versus* the orientation of the two side phenyl ring relative to the plane of the two terminated phenyl rings connected to SMe anchor group for [5]cumulene.

The [3] and [5]cumulenes are synthesized as inseparable mixtures of *E* and *Z* isomers. We tested whether the *E*/*Z* stereochemistry would be expected to have a strong effect on the conductance. Taking [3]cumulene as an example, Figure S7 shows a comparison between the transmission coefficients of the *E* and *Z* isomers and reveals that the difference between them is negligible.

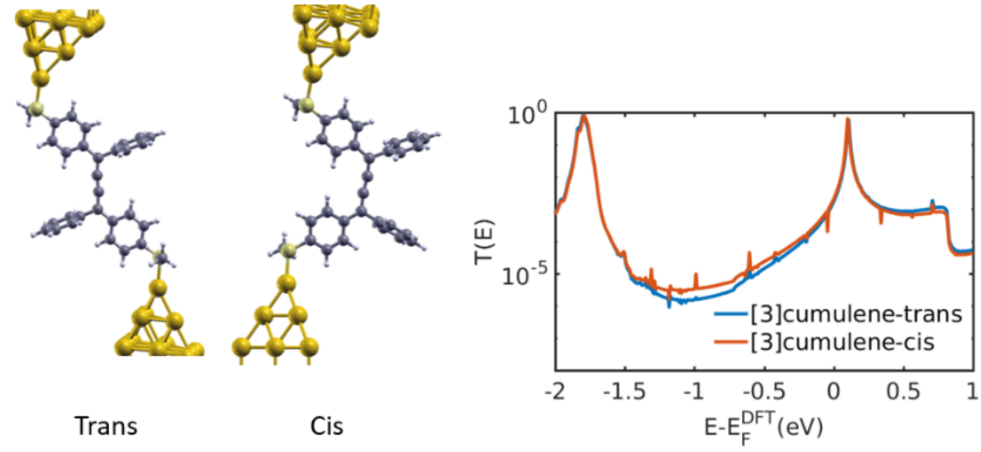

Figure S7. Transmission functions of two stereoisomers (*E*/*trans* and *Z*/*cis*) of the [3]cumulene.

The data reported by Tykwinski and coworkers in ref. [17] indicate that the barriers to rotation in [3] and [5]cumulenes with Ph substituents are 23.59 and 15.63 kcal mol^{-1}, respectively. These barriers correspond to exchange rates of 3.2×10^{-5} s^{-1} and 22 s^{-1}, respectively, which correspond to half-lives of 6.0 hours and 32 ms, respectively, at 298 K. Thus we expect the timescale for thermal interconversion of the *E* and *Z* isomers is shorter than the time required to carry out single-molecule conductance measurements.

Section S3. STM Break-Junction Measurements

S3.1. Sample Preparation for Single-Molecule Experiments. Each compound was deposited onto a freshly flame-annealed gold substrate from a 0.1–1.0 mM dichloromethane solution, using an immersion time of 5 minutes. After this time, the substrate was removed and blown dry. To prepare the tip, we mechanically cut a gold wire, rinse it with ethanol to remove any grease and then pass it briefly through a butane flame.

The samples used for our STM single-molecule conductance measurements were prepared by removing all solvent before mounting in the microscope. Thus our results should be considered as solvent-free. These are the standard conditions that we have employed for many other systems, such as the widely studied oligo(phenylene ethynylene) – OPE – compounds, and thus they provide a baseline environment for comparison of different compounds. Typically, excellent agreement is found for measurements performed between different labs (see, for example, data for a series of OPE-TTF compounds measured under ambient with an STM and an MCBJ; ref. [18]). Our previous results suggest that, for neutral OPEs molecules, solvent-molecule interactions are negligible as far as the BJ method is concerned;[19] we see no reason for cumulenes to behave differently. The polymethine systems studied by Venkataraman and coworkers are cationic molecules and solvent effects are expected to be more significant with these systems.[20]

S3.2. Single-Molecule Conductivity Studies. The conductance of each compound was measured using the STM-BJ method. All experiments were performed using a home-built STM, optimized for room temperature measurements in air. During the break-junction experiment, the tip is moved vertically in and out of contact with the substrate at a constant speed of approximately 10 nm/s, in 1 pm steps. For the conductance ($G = I/V$) *versus* distance measurements, a bias voltage V of 0.2 V was applied between the tip and the substrate. A linear current-to-voltage converter with two amplification stages allows us to record conductance values over a large dynamic range. The gains used in this study were 5×10^7 V A^{-1} and 2.4×10^9 V A^{-1}. We also place a resistor in series with the circuit of 1 MΩ.

The motion of the tip and the conductance measurement are controlled by an in-house computer program to record conductance *versus* tip-displacement (G *vs.* z) curves. Typically, when moving out of contact, we move several nm after reaching our lower conductance limit. When in contact, the piezo moves a further 1–2 nm after reaching 1.0 G_0. These limits guarantee that a broad gold contact is formed and broken in each cycle, and that any molecular junction is broken at the end of the cycle. We aim to carry out 5000–10000 approach and retraction cycles in each run, and we vary the location of the tip over the sample in order to avoid systematic errors in the data. We focus on the opening stages of the cycle. After data acquisition is complete, we first remove any blocks of traces in which there is a clear degradation in trace quality due (most likely) to tip blunting or excessive build-up of material between the electrodes. We then use an automated routine to separate traces displaying plateaus from those with tunneling-only. This searches for regions of individual traces in which the conductance change is less than a certain amount across a minimum distance interval (for example, a plateau is identified when a $\Delta z > 0.1$ nm is needed to produce a change in conductance of $\Delta\log(G/G_0) < 0.1$ at any region below 0.5 G_0). We aim to use very similar criteria for all compounds.

S3.3. 2D Histograms

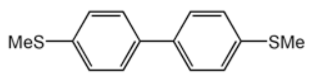

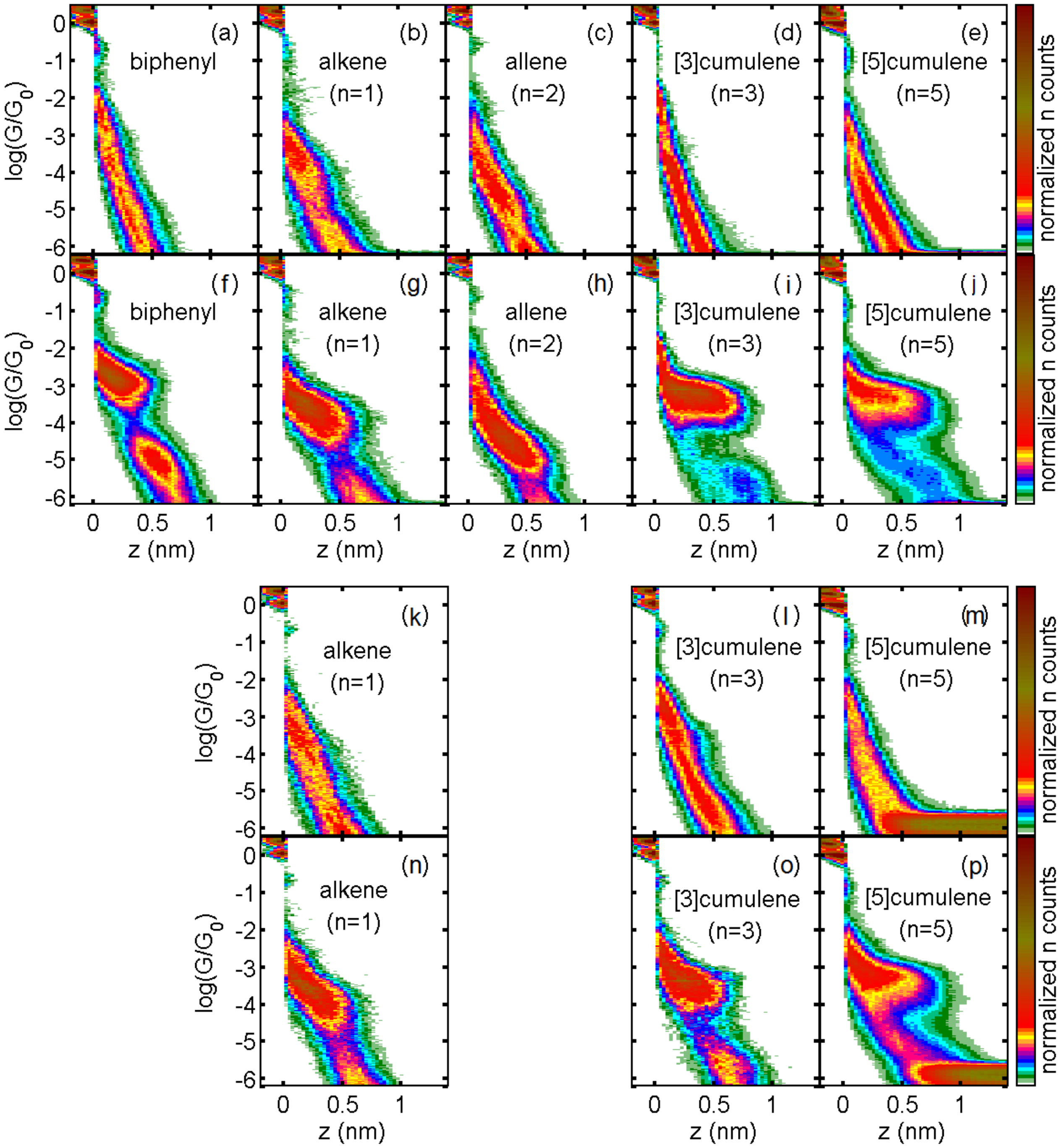

Figure S8. 2D histograms for all experimental runs carried out for bis-4,4'-(thiomethyl)biphenyl, alkene, allene, [3]cumulene and [5]cumulene showing both 'molecule-free' and molecular junctions. In some cases, the faint second (lower) conductance group is probably due to π-stacked molecules. For the biphenyl, this was quite strong in most of the measurement. The number of traces in each category, and the corresponding percentages of the overall number, are given in the Table S1.

Compound	Molecule-free junctions	Molecular junctions
biphenyl	(a) 1189 (26%)	(f) 3404 (74%)
alkene (run 1)	(b) 1037 (44%)	(g) 1309 (56%)
alkene (run 2)	(k) 844 (44%)	(n) 1075 (56%)
allene	(c) 2198 (42%)	(h) 2988 (58%)
[3]cumulene (run 1)	(d) 1286 (53%)	(i) 1139 (47%)
[3]cumulene (run 2)	(l) 3779 (84%)	(o) 708 (16%)
[5]cumulene (run 1)	(e) 6718 (66%)	(j) 3402 (34%)
[5]cumulene (run 2)	(m) 1780 (32%)	(p) 3712 (68%)

Table. S1. Percentages of junctions with and without molecules for each experimental run.

S3.4. Plateau Length Distributions

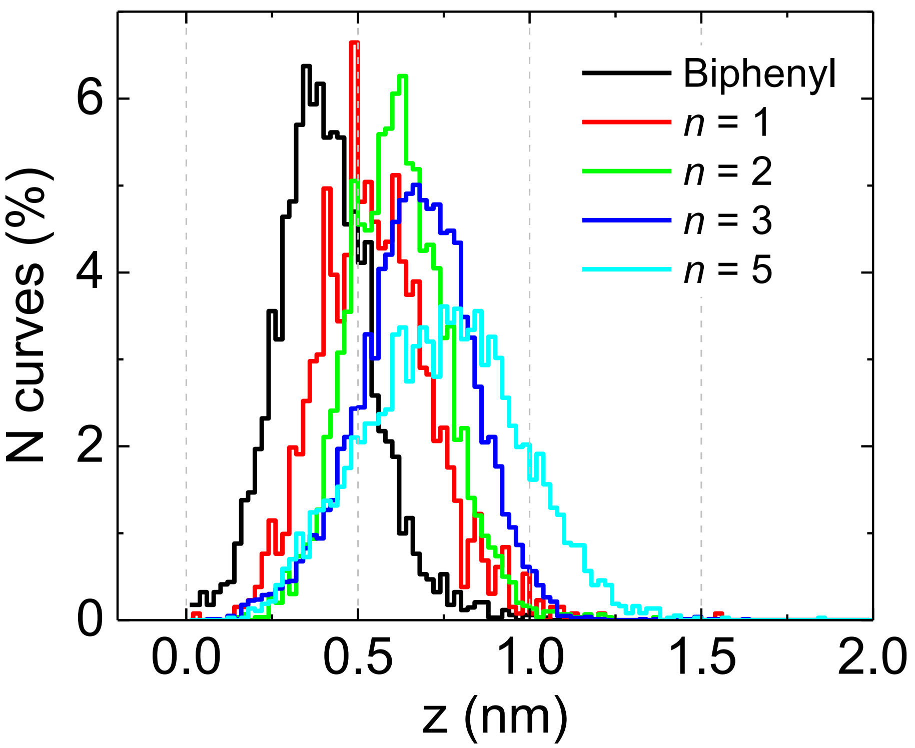

Figure S9. Histogram of plateau length distributions for run 1 of each compound (these are the measurements presented in Figure 2 of the main paper). The distributions are determined by measuring the distance interval between two conductance values in each trace. The first is at a value of $\log(G/G_0) = 0.5$, and the second value is chosen to be slightly below the peak position of the 1D conductance histogram. These data are the raw values before calibration.

S3.5. Voltage Dependence of Molecular Conductance

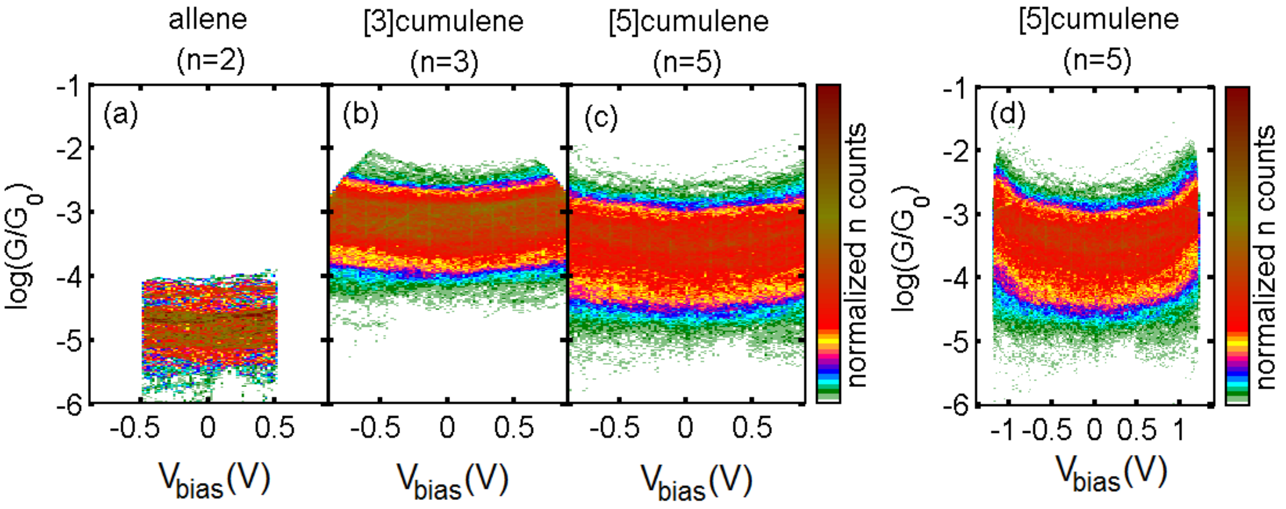

Figure S10. Plot showing conductance as a function of bias voltage for (a) [2]cumulene, (b) [3]cumulene and (c) [5]cumulene. The number of *I-V* curves/molecular junctions used to generate each histogram were as follows: (a) 182/40, (b) 1561/79, (c) 1504/350. For the allene or [2]cumulene, it was not possible to measure much beyond 0.5 V, whereas with the [5]cumulene we could increase the bias voltage to 1.2 V before the junctions start to become unstable (d). Plot (d) contains the same data as plot (c), just to show the entire range measured.

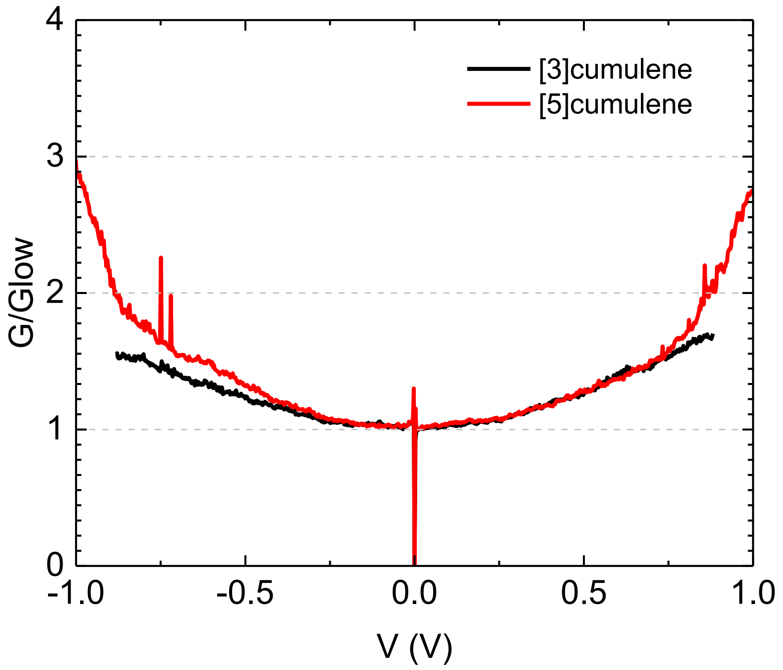

Figure S11. Plot of the normalized conductance for [3] and [5]cumulenes, obtained by dividing each *G-V* trace by the low-bias conductance and then averaging. This shows that at a bias of 1.0 V, [5]cumulene has a conductance three times greater than at 0 V. This degree of change is comparable with a porphyrin monomer that we tested recently, but much less than for the series of fused porphyrin oligomers.[21]

S3.6. Current through [5]Cumulene

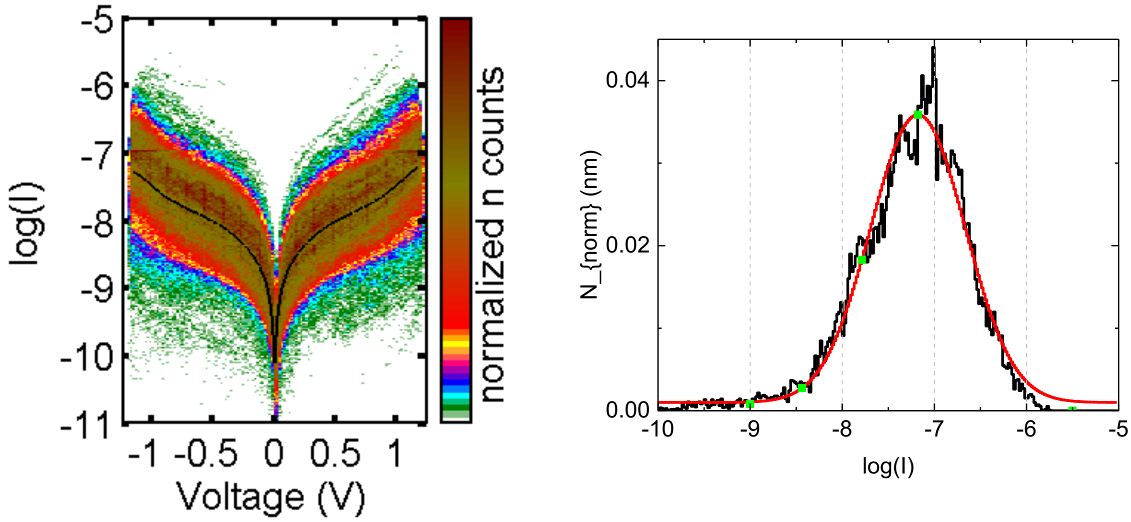

Figure S12. (Left) 2D histogram of the current measured for [5]cumulene for each trace. The black line is the mean of the log(I). (Right) 1D histogram of a small portion of the data between +1.10 and +1.15 V. The most probable current in this range is given by the Gaussian fit, yielding a median value of 0.06 μA.

S3.7. Bis-4,4'-(thiomethyl)biphenyl

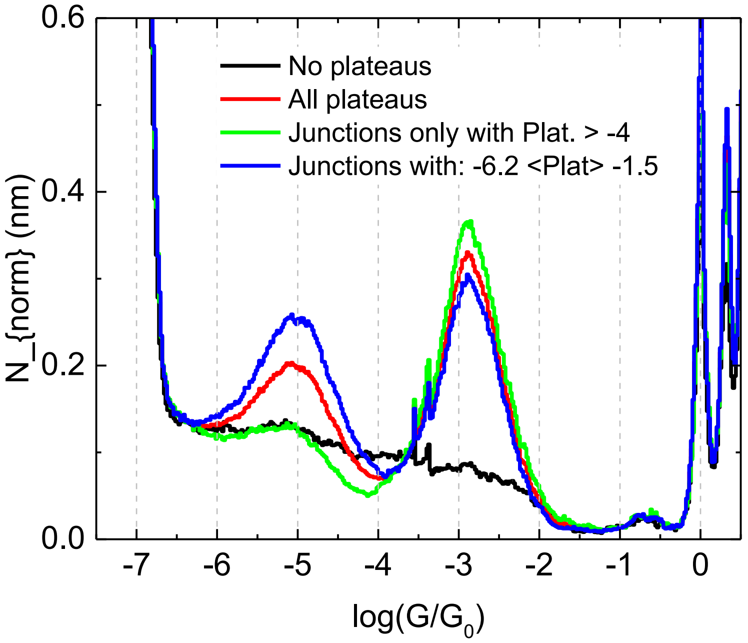

Figure S13. 1D conductance histograms for the biphenyl compound. Two conductance groups can be seen in the total histogram with molecular junctions (red). The higher group can be attributed to the 'single molecule' junctions, whilst the lower can be ascribed to π-stacked junctions.[22,23] The high percentage of plateaus relative to molecule-free junctions (74%) indicates that there are many molecules in the vicinity of the junction, making multi-molecular junctions plausible. The number of traces in each histogram are as follows; 'No plateaus' (black): 1189, 'All plateaus' (red): 3404, 'Junctions with only Plat. > log(G/G_0) = –4 (green): 1448, 'Junctions with –6.2 < Plat. > –1.5 (blue): 1956.

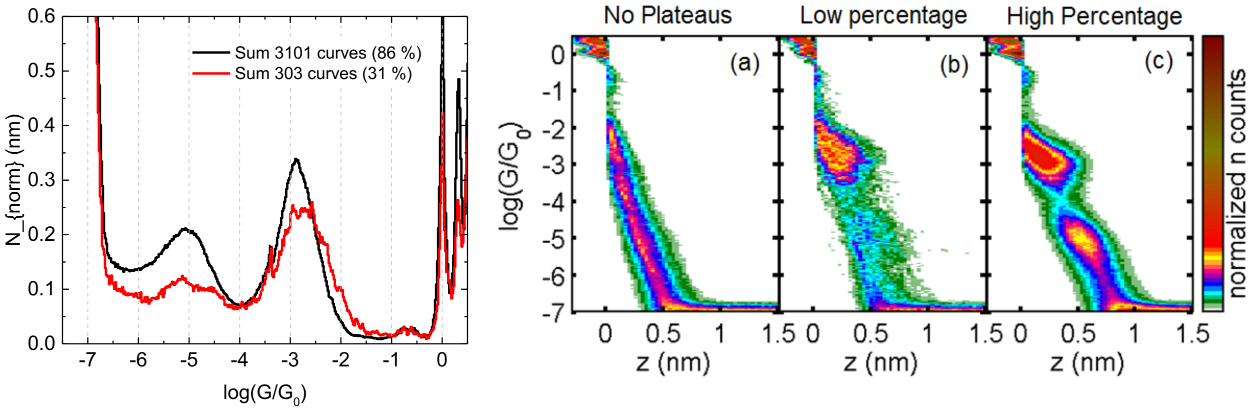

Figure S14. (Left) 1D histograms of the biphenyl during periods of different transient molecular junction percentages (black – high percentage period, red – low percentage period). (Right) 2D histograms of the different measurement periods. The low conductance peak at roughly $\log(G/G_0) = -5$ is weaker when the percentage is low, fitting with this group originating from multiple 'π'-stacked molecules. Unlike for the longer cumulene compounds (see Section S3.8), despite the significantly higher percentage of molecular junctions, the main peak at about $\log(G/G_0) = -3$ remains centered essentially in the same position during both periods. The high percentage data can be rationalized by considering that despite there being more molecules in the vicinity of the junction, so the overall binding frequency is higher, binding more molecules in parallel is difficult due to their short length. The tip is expected to be convex, so there is a smaller area (fewer binding sites) for shorter molecules to bridge compared to longer molecules.

S3.8. High *versus* Low Percentages of Molecular Junctions

Molecules with weak anchor groups are prone to giving high percentages of plateaus, making junctions more likely to contain multiple molecules. Between different experimental runs, the percentage of molecular junctions can vary significantly, most likely reflecting local density differences in molecular coverage and/or different surface profiles. Both runs for [3]cumulene, for example, yielded molecular junction percentages of 47% and 16% respectively. 1D conductance histograms of the two data sets (Figure S15b) show that the peak of the higher percentage set is clearly weighted to higher conductance values. Run 1 of the [5]cumulene yielded an overall percentage of molecular junctions of 34%, but this does not tell the full picture. During a single experimental run (which takes many hours to complete) it is sometimes possible to find significant variation in the transient percentage of molecular junctions. To analyze this for the [5]cumulene we broke the measurement down into blocks of 500 traces, and looked at the percentage of molecular junctions for each block. Previous work on oligo(phenylene diamines)[19] has shown that stable (and therefore reliable) single molecule conductance values can be obtained when the percentage of molecular junctions is below about 40%. Hence we divided the traces into two groups, those with a transient percentage greater/lower than 40%. From the black (< 40%) and the red (> 40%) histograms in Figure S15c it is clear that the lower percentage data from within the same run yield a lower maximum peak. The overall percentage of plateaus in each group (determined by averaging the percentages for each 500 trace block) were 51% for the '> 40%' set and 19% for the '< 40%' set.

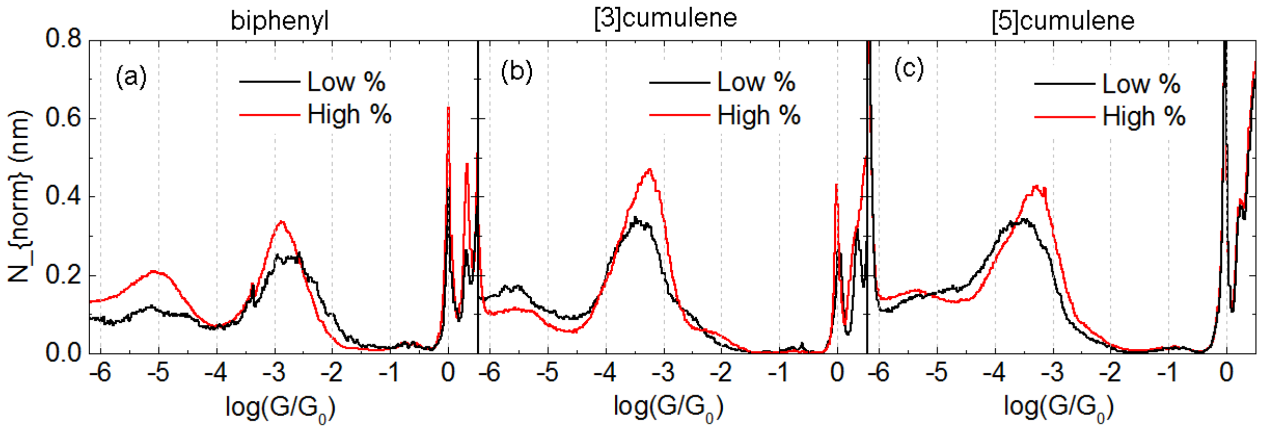

Figure S15. High *versus* low percentages of molecular junctions found during the measurements. For the biphenyl (a) and the [5]cumulene (c) the different percentages were observed during the same run (*i.e.* on the same sample). For the [3]cumulene (b) these differences were only found between two separate runs (*i.e.* on different samples), with the 'high' % data coming from run 1, and the 'low' % from run 2.

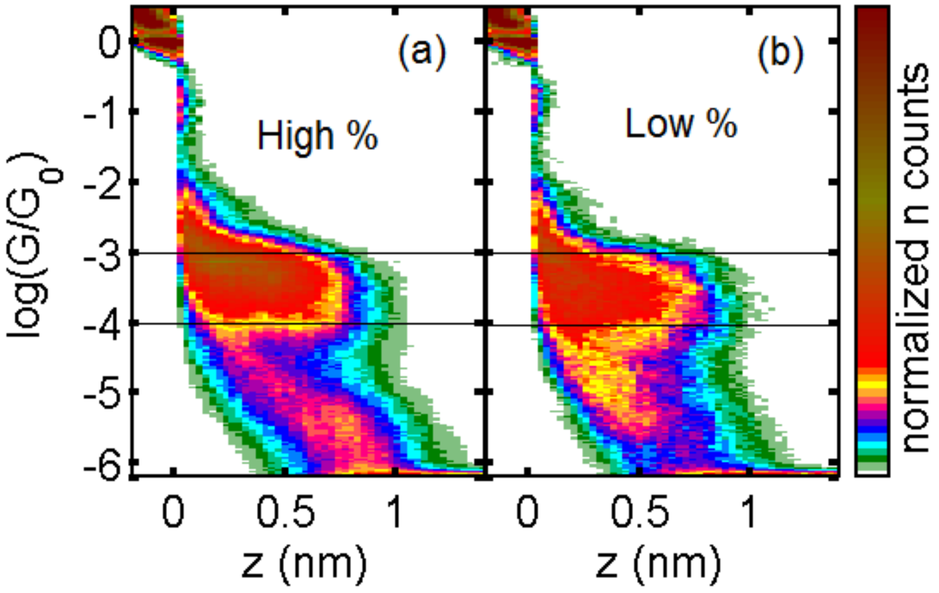

Figure S16. 2D histograms of the high (a) and low (b) percentage segments of run 1 for [5]cumulene respectively. The horizontal lines are guides for eye only.

S3.9. Measuring the Conductance at the End of the Plateau Length Distribution

Taking the [5]cumulene data first, if we now restrict the points in the histograms to only those towards the end of the distributions, we see that the resultant histograms overlap very well (Figure S18b). The procedure we have used to do this is first to fit the length distribution histograms (Figure S18a) with a single Gaussian to obtain a peak position (*i.e.* the most frequent break-off distance). For the [5]cumulene this was 0.68 nm (uncalibrated value). We then select only points greater than this distance from the total data set of traces and use these to plot the final histograms (as shown in Figure 3f in the main paper). Apart from removing many points from the traces, this also means that junctions with plateaus shorter than 0.68 nm will not contribute to these histograms, reducing the overall number of contributing junctions.

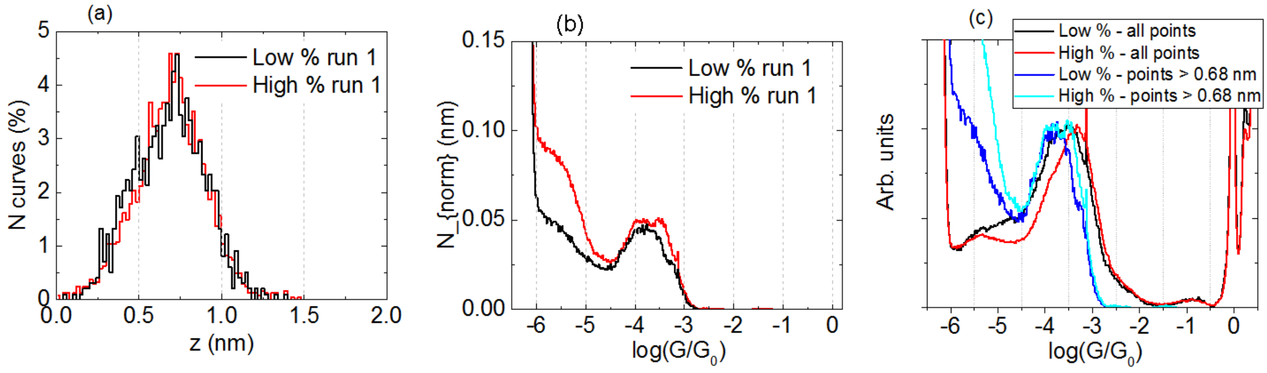

Figure S17. Measuring at the end of the plateau distribution for [5]cumulene. (a) Plateau length distributions for the high and low percentage regions. (b) 1D conductance histograms of the high and low percentage data points beyond 0.68 nm. (c) All-data point and 'restricted' histograms normalized to a common peak height.

We have also carried out the same procedure for the two separate runs of the [3]cumulene, which gave different percentages of molecular junction. The resulting histograms in Figure S18 show that again good agreement is found between the two runs when considering only the final points in the plateau distribution. Quantifying the conductance in this way is the best we can do to ensure we have the true conductance of a fully stretched single molecule.

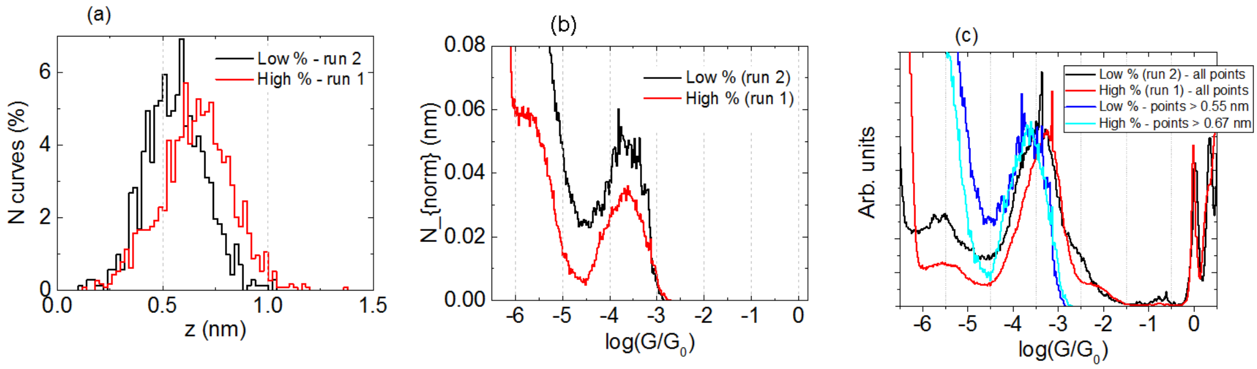

Figure S18. Measuring at the end of the plateau distribution for [3]cumulene. (a) Plateau length distributions for the high and low percentage runs. (b) 1D conductance histograms of the high and low percentage data points beyond 0.55 nm for run 2 and 0.67 nm for run 1. (c) All-data point and 'restricted' histograms normalized to a common peak height.

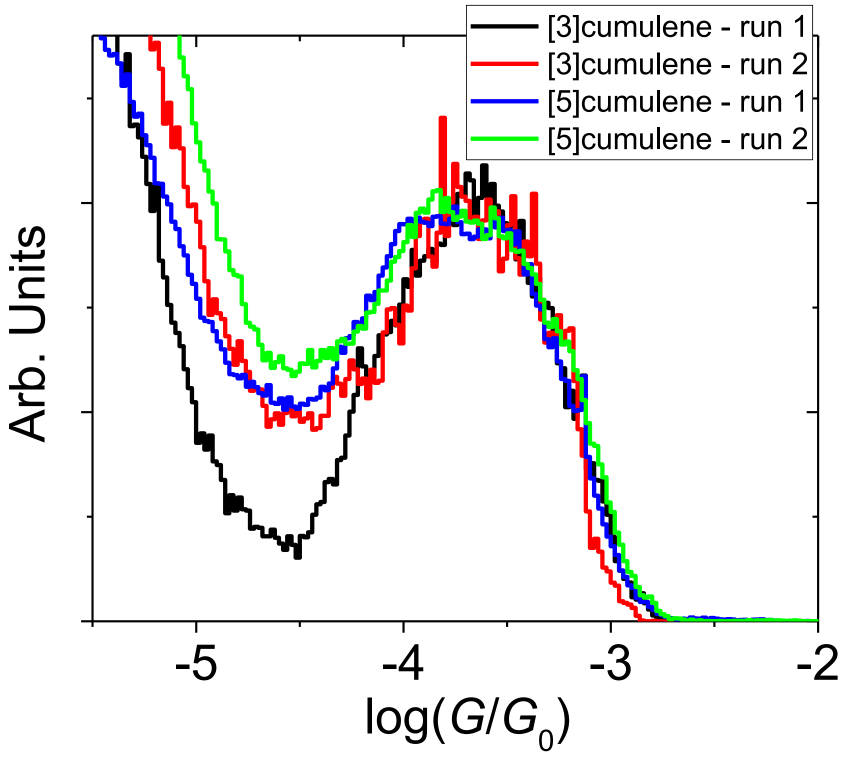

Figure S19. Comparison of the 1D conductance histograms of [3] and [5]cumulenes at the extreme of the distribution over the two experimental runs for each compound. All histograms have been normalized to the same peak height. The figure highlights the very good reproducibility between experimental runs, and seems to suggest a slightly broader conductance distribution of the [5]cumulene, weighted to lower conductance values. The centres of Gaussian fits to each are located at: [3]cumulene – run 1: $\log(G/G_0) = –3.70$, [3]cumulene – run 2: $\log(G/G_0) = –3.70$, [5]cumulene – run 1: $\log(G/G_0) = –3.79$, [5]cumulene – run 2: $\log(G/G_0) = –3.77$.

Section S4. NMR Spectra of New Compounds

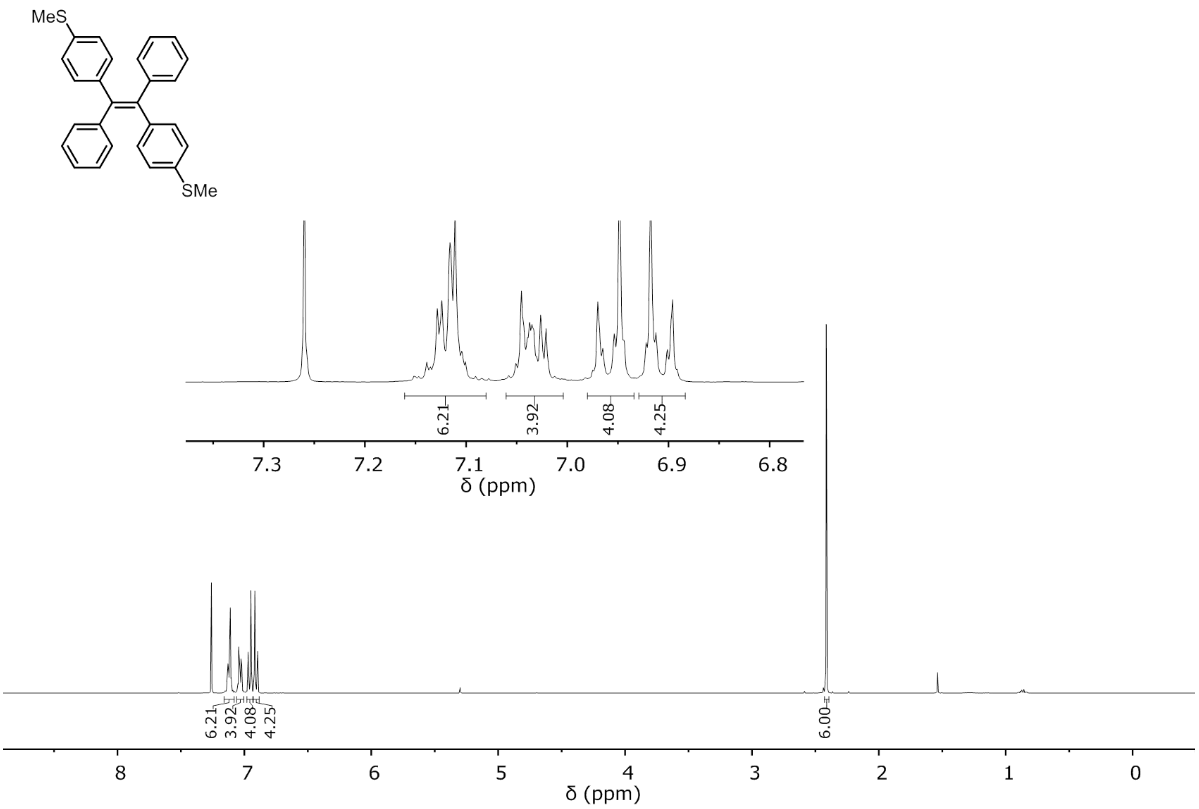

Figure S20. ^{1}H NMR spectrum of 1,2-bis(4-(methylthio)phenyl)-1,2-diphenylethene **1** ($CDCl_3$, 400 MHz, 298 K).

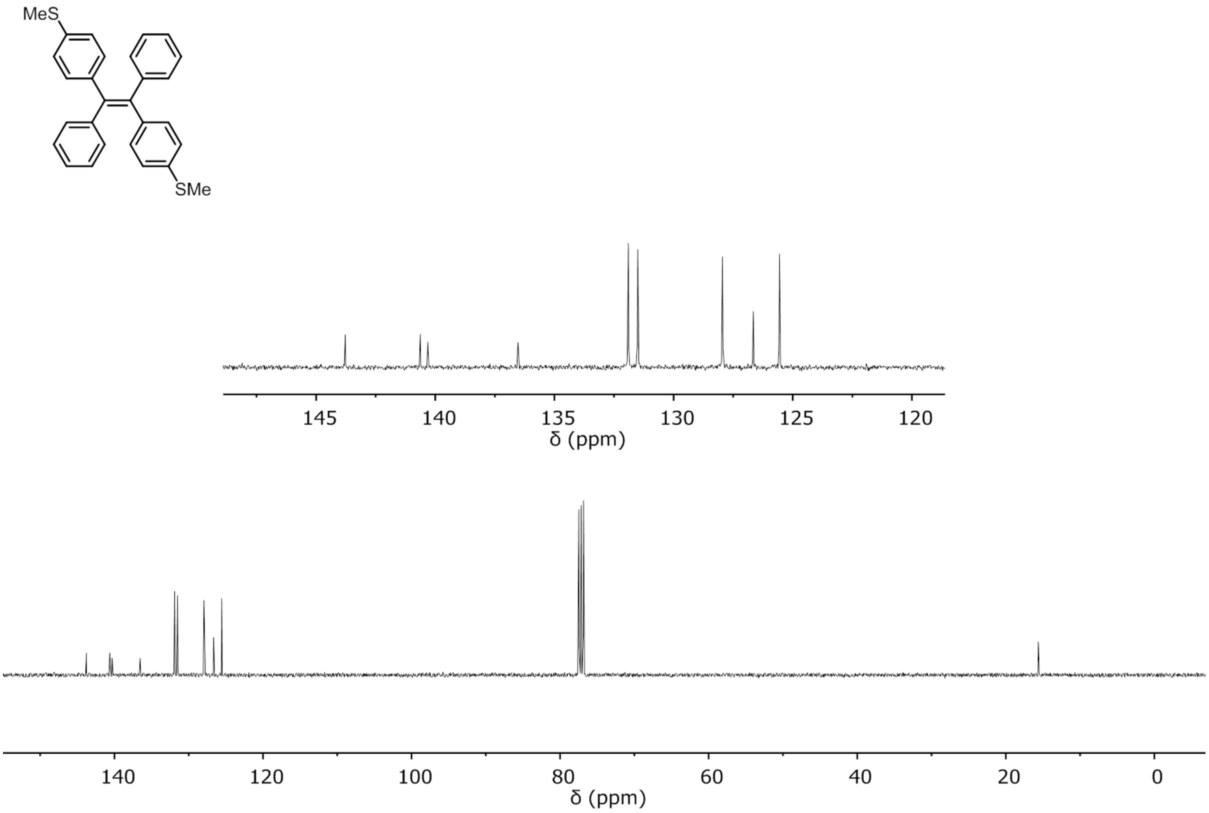

Figure S21. ^{13}C NMR spectrum of 1,2-bis(4-(methylthio)phenyl)-1,2-diphenylethene **1** ($CDCl_3$, 100 MHz, 298 K).

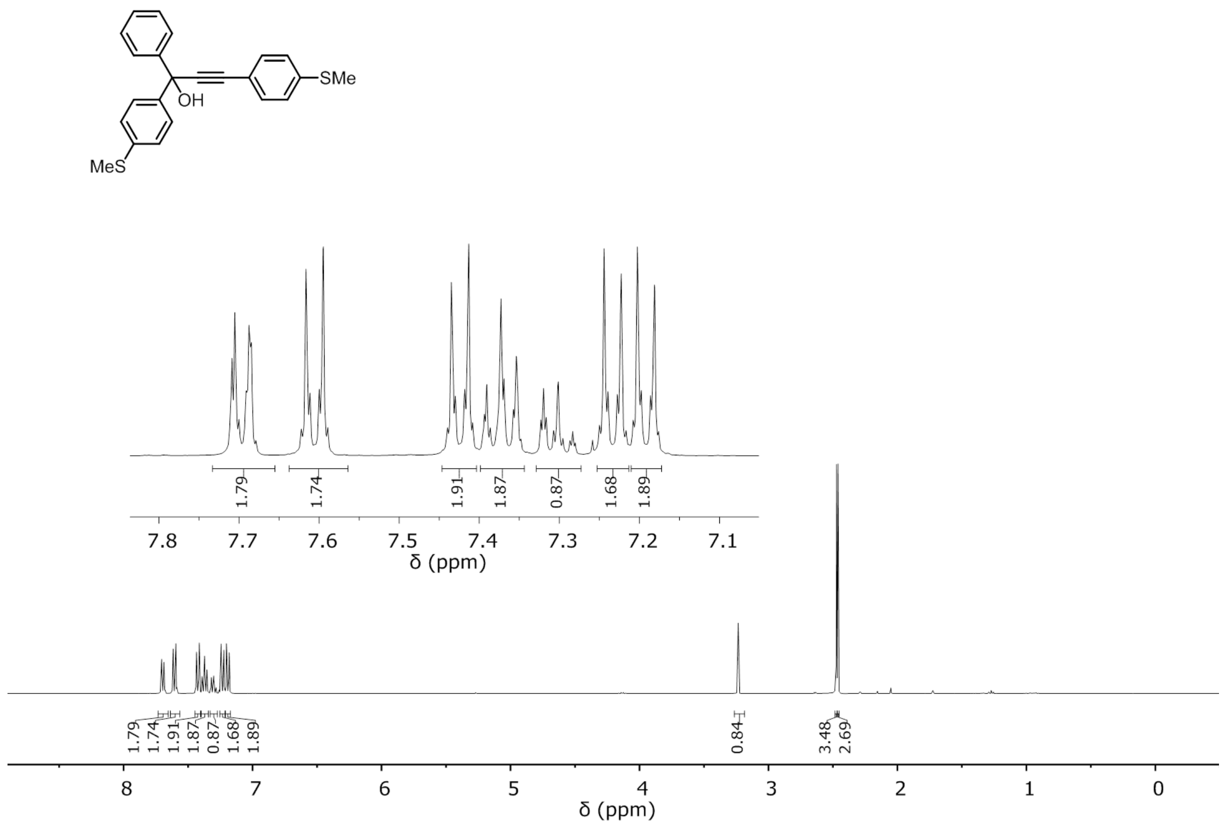

Figure S22. ^{1}H NMR spectrum of 1,3-bis(4-(methylthio)phenyl)-1-phenylprop-2-yn-1-ol **8** ($CDCl_3$, 400 MHz, 298 K).

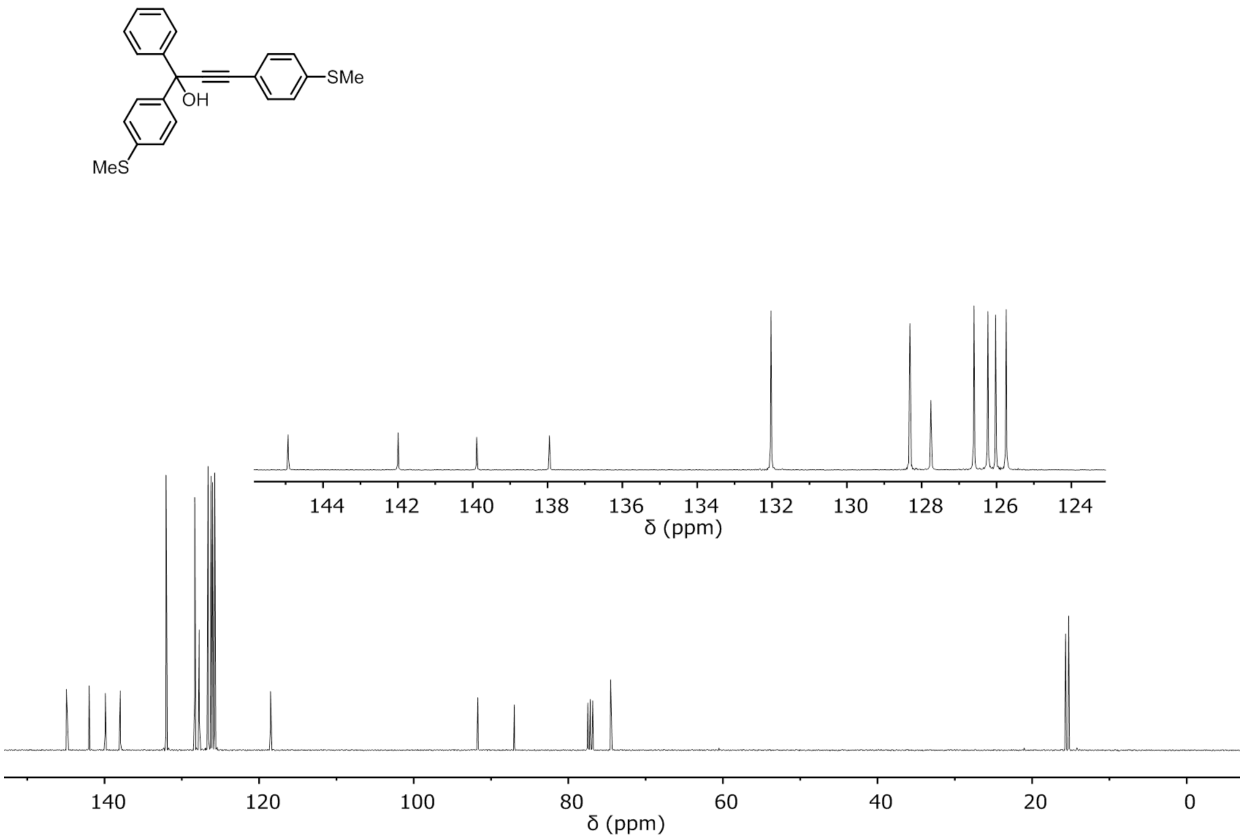

Figure S23. ^{13}C NMR spectrum of 1,3-bis(4-(methylthio)phenyl)-1-phenylprop-2-yn-1-ol **8** ($CDCl_3$, 100 MHz, 298 K).

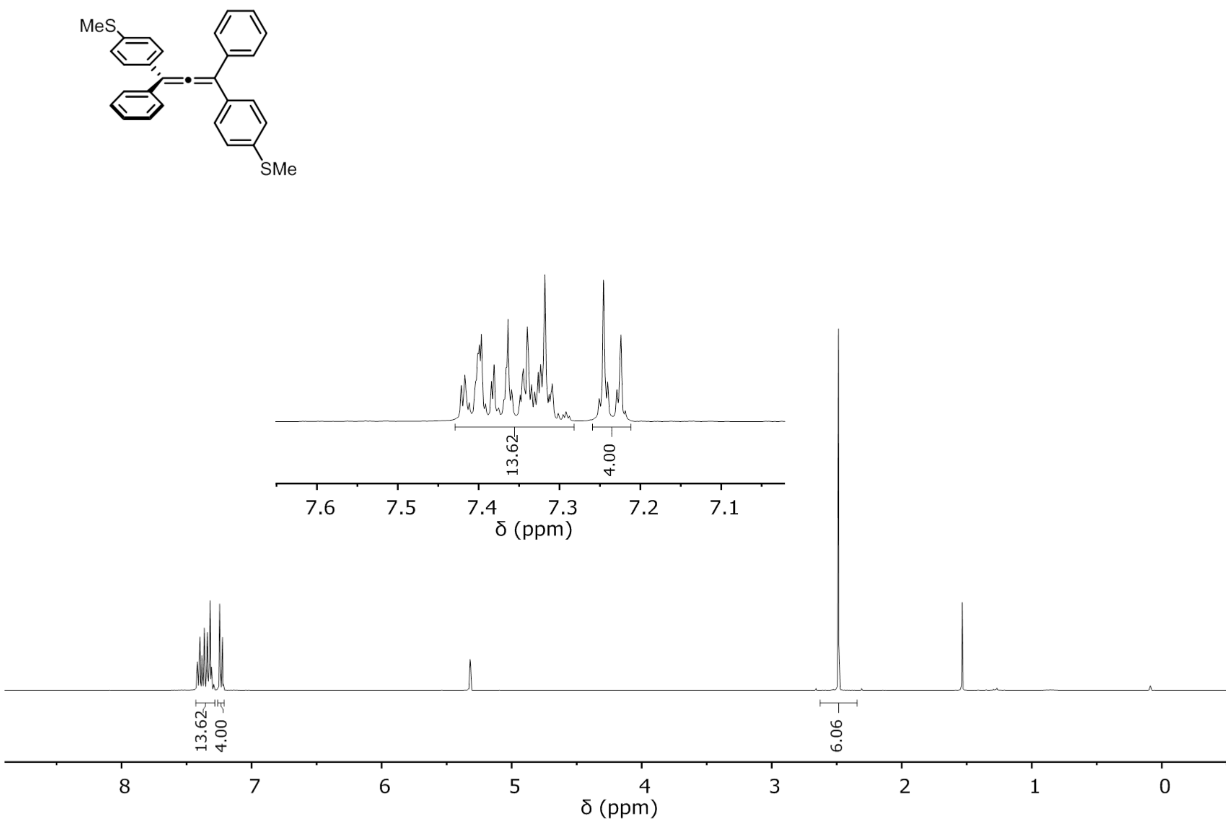

Figure S24. ^{1}H NMR spectrum of 1,3-bis(4-(methylthio)phenyl)-1,3-diphenylpropa-1,2-diene **2** (CD_2Cl_2, 400 MHz, 298 K).

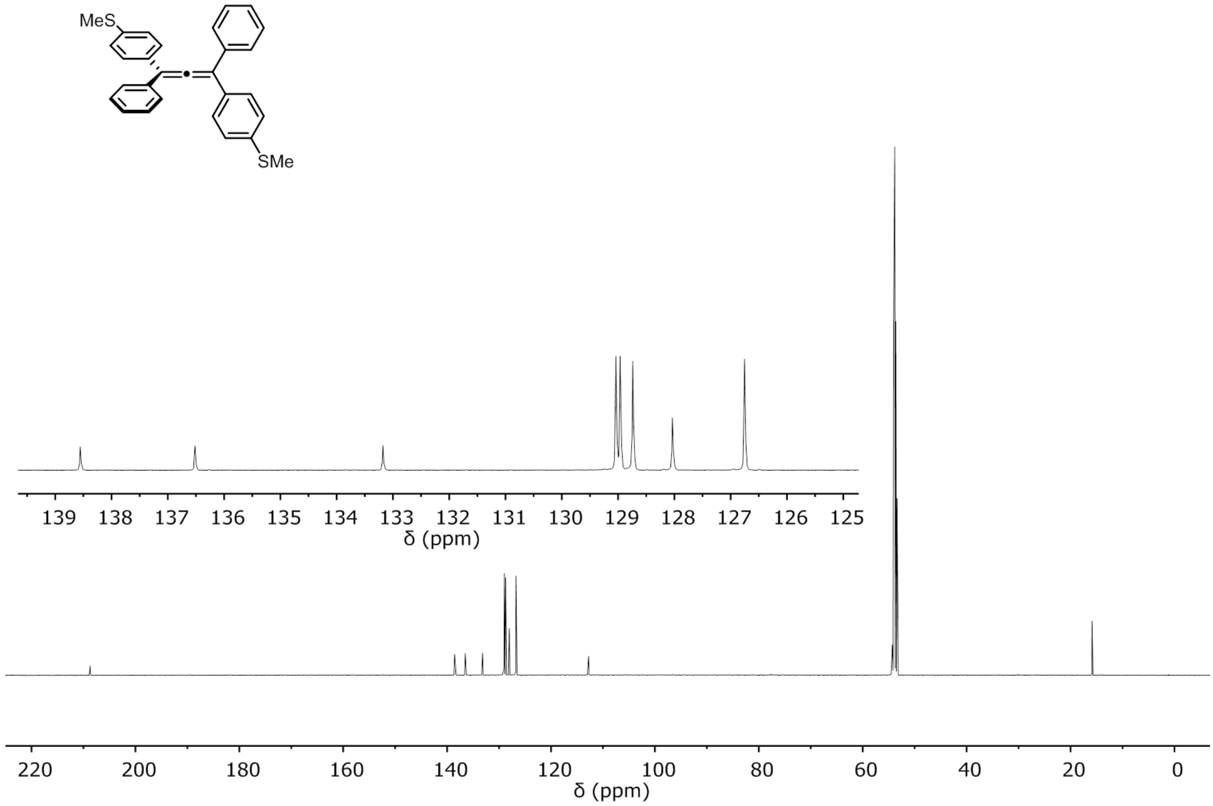

Figure S25. ^{13}C NMR spectrum of 1,3-bis(4-(methylthio)phenyl)-1,3-diphenylpropa-1,2-diene **2** (CD_2Cl_2, 125 MHz, 298 K).

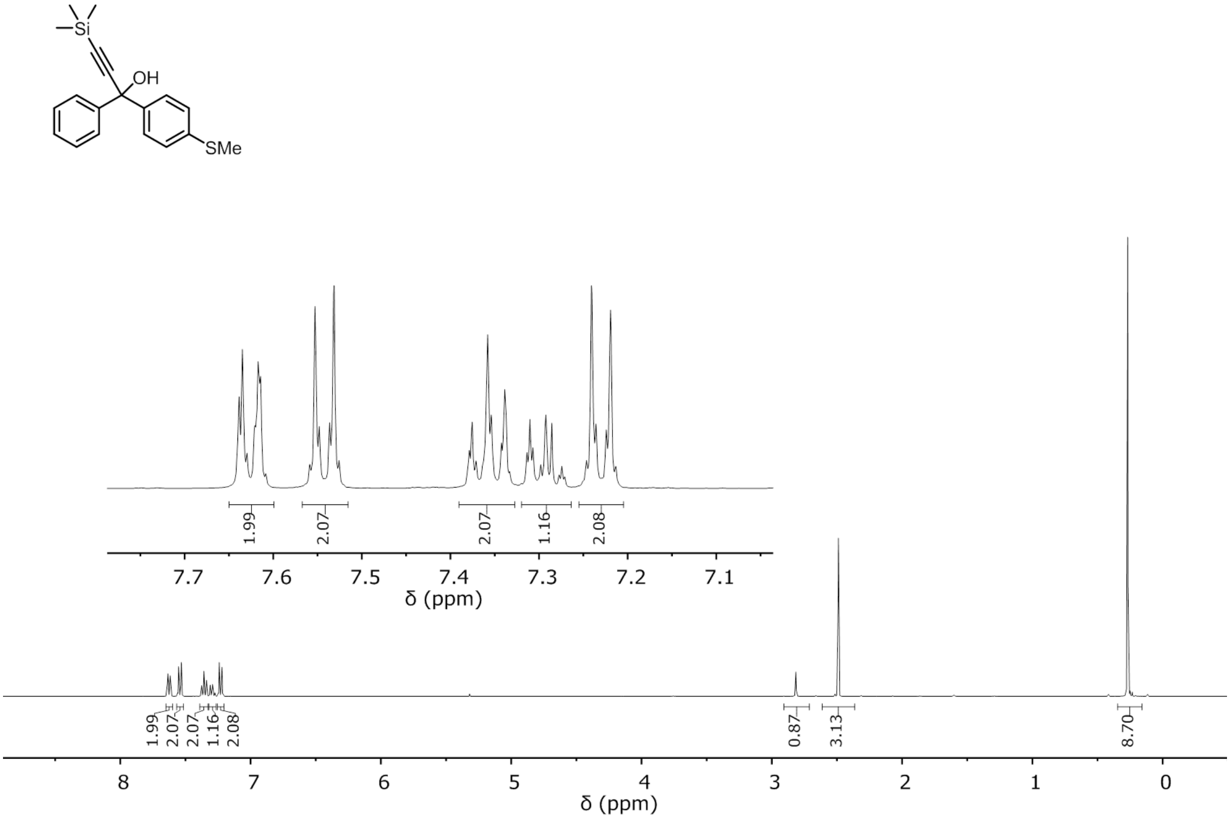

Figure S26. ^{1}H NMR spectrum of 1-(4-(methylthio)phenyl)-1-phenyl-3-(trimethylsilyl)prop-2-yn-1-ol **9** ($CDCl_3$, 400 MHz, 298 K).

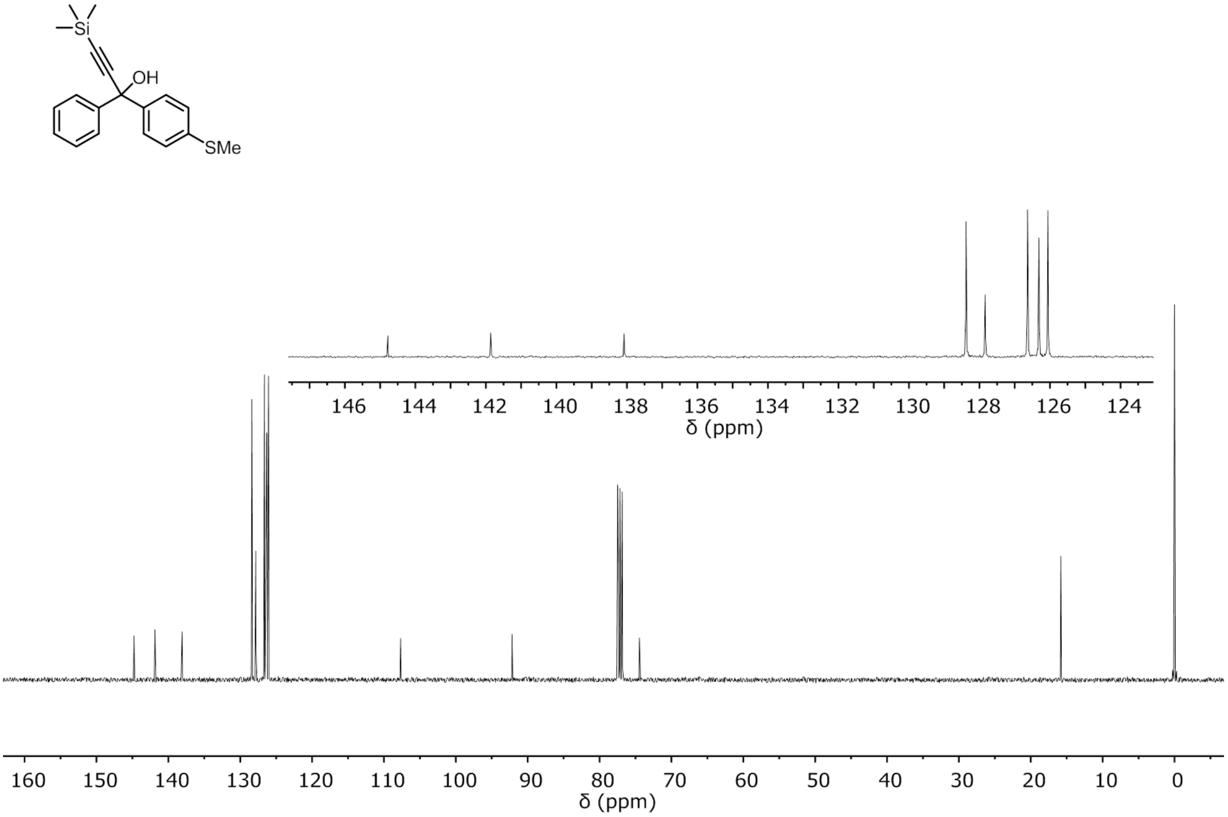

Figure S27. ^{13}C NMR spectrum of 1-(4-(methylthio)phenyl)-1-phenyl-3-(trimethylsilyl)prop-2-yn-1-ol **9** ($CDCl_3$, 100 MHz, 298 K).

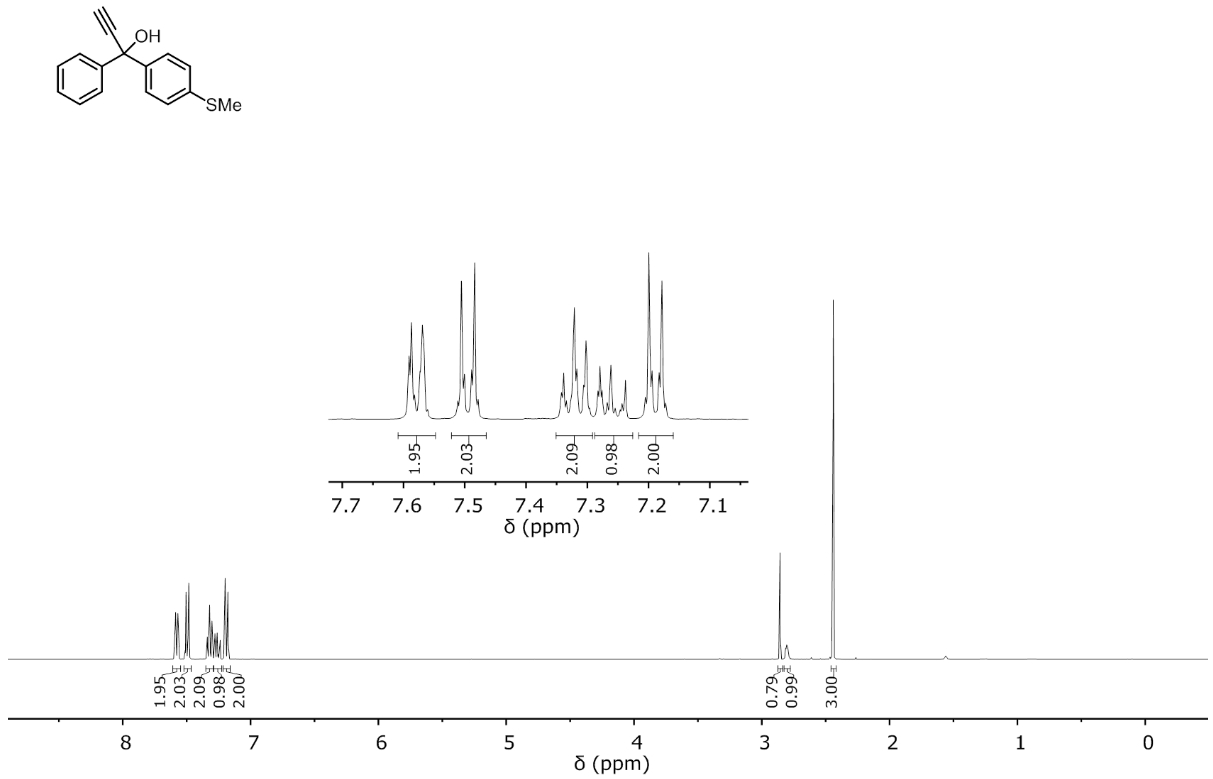

Figure S28. ^{1}H NMR spectrum of 1-(4-(methylthio)phenyl)-1-phenylprop-2-yn-1-ol **10** ($CDCl_3$, 400 MHz, 298 K).

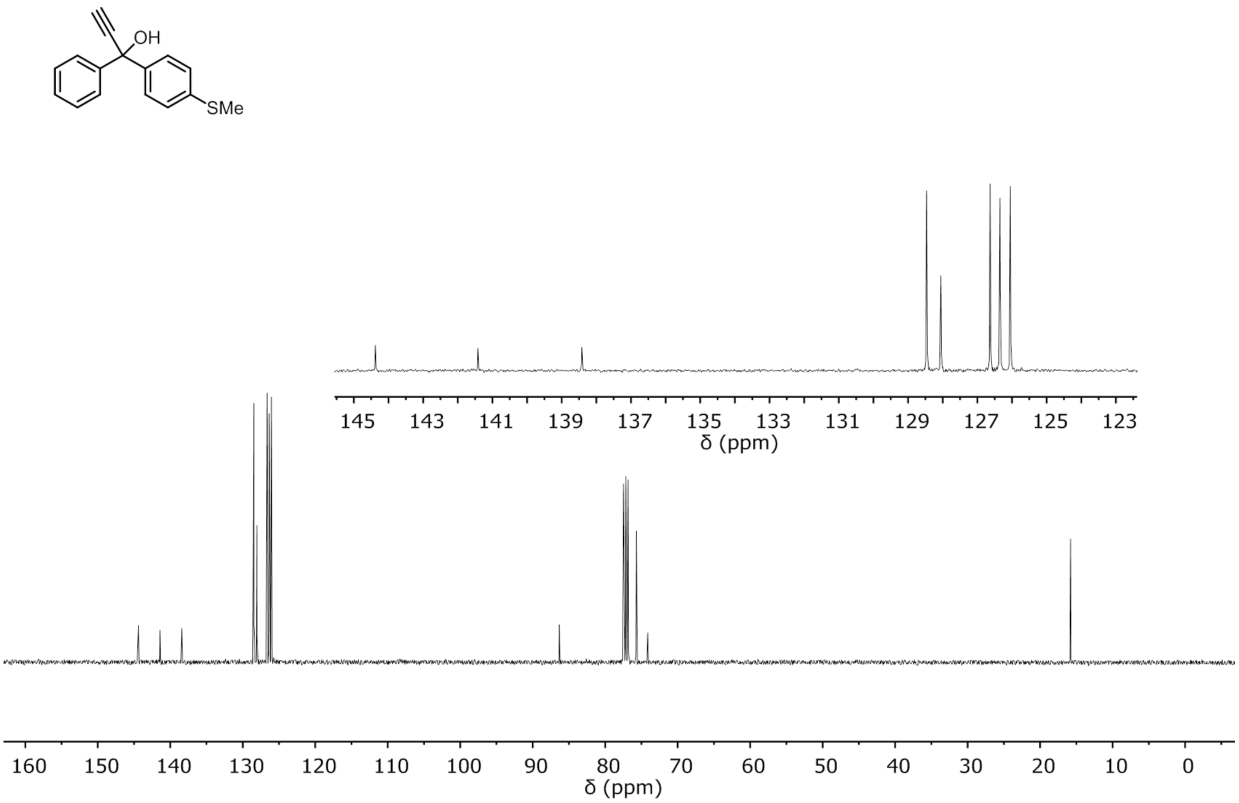

Figure S29. ^{13}C NMR spectrum of 1-(4-(methylthio)phenyl)-1-phenylprop-2-yn-1-ol **10** ($CDCl_3$, 100 MHz, 298 K).

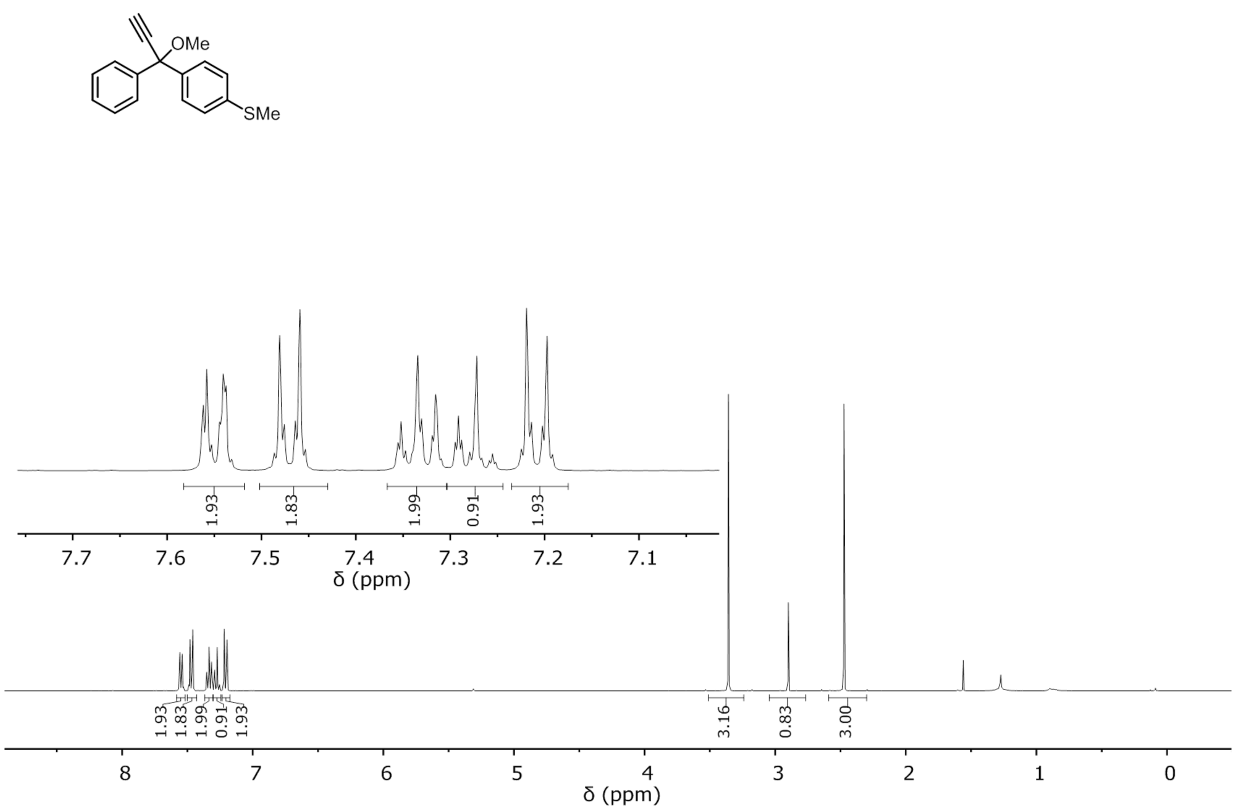

Figure S30. ^{1}H NMR spectrum of (4-(1-methoxy-1-phenylprop-2-yn-1-yl)phenyl) (methyl)sulfane **11** ($CDCl_3$, 400 MHz, 298 K).

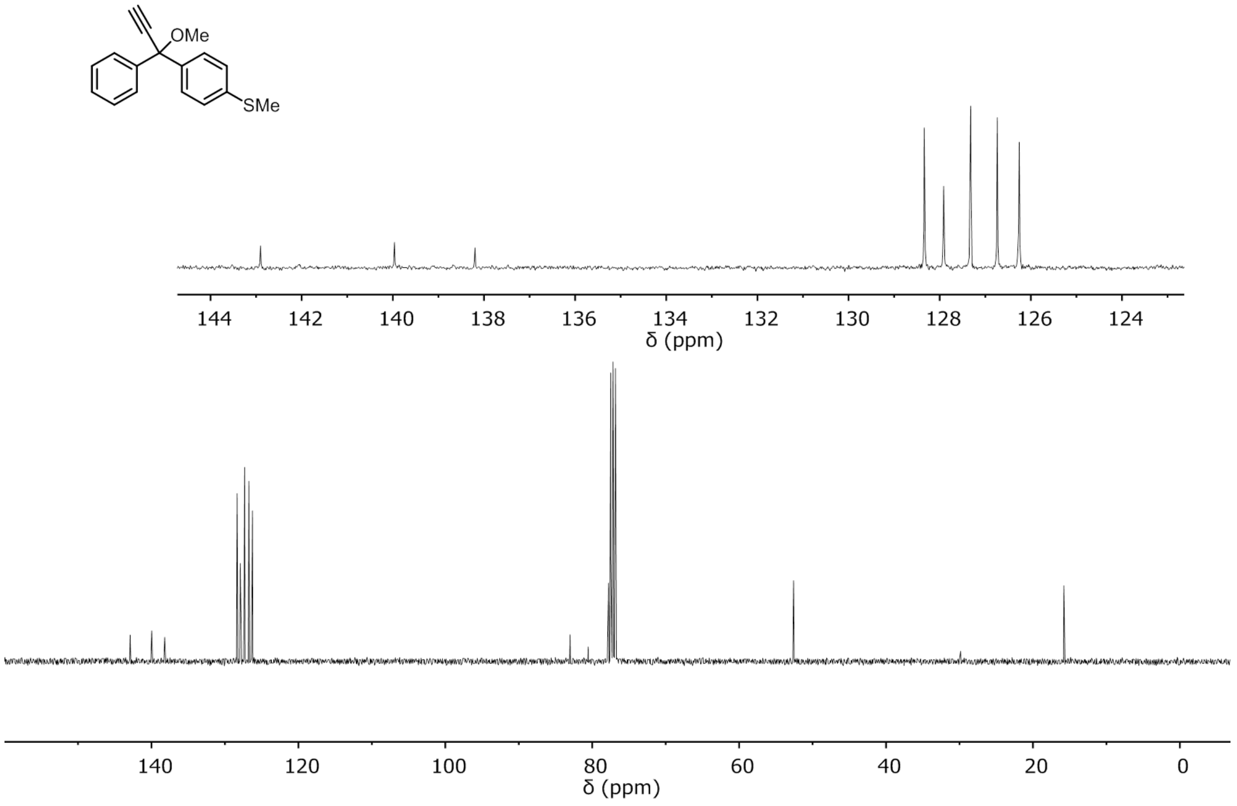

Figure S31. ^{13}C NMR spectrum of (4-(1-methoxy-1-phenylprop-2-yn-1-yl)phenyl) (methyl)sulfane **11** ($CDCl_3$, 100 MHz, 298 K).

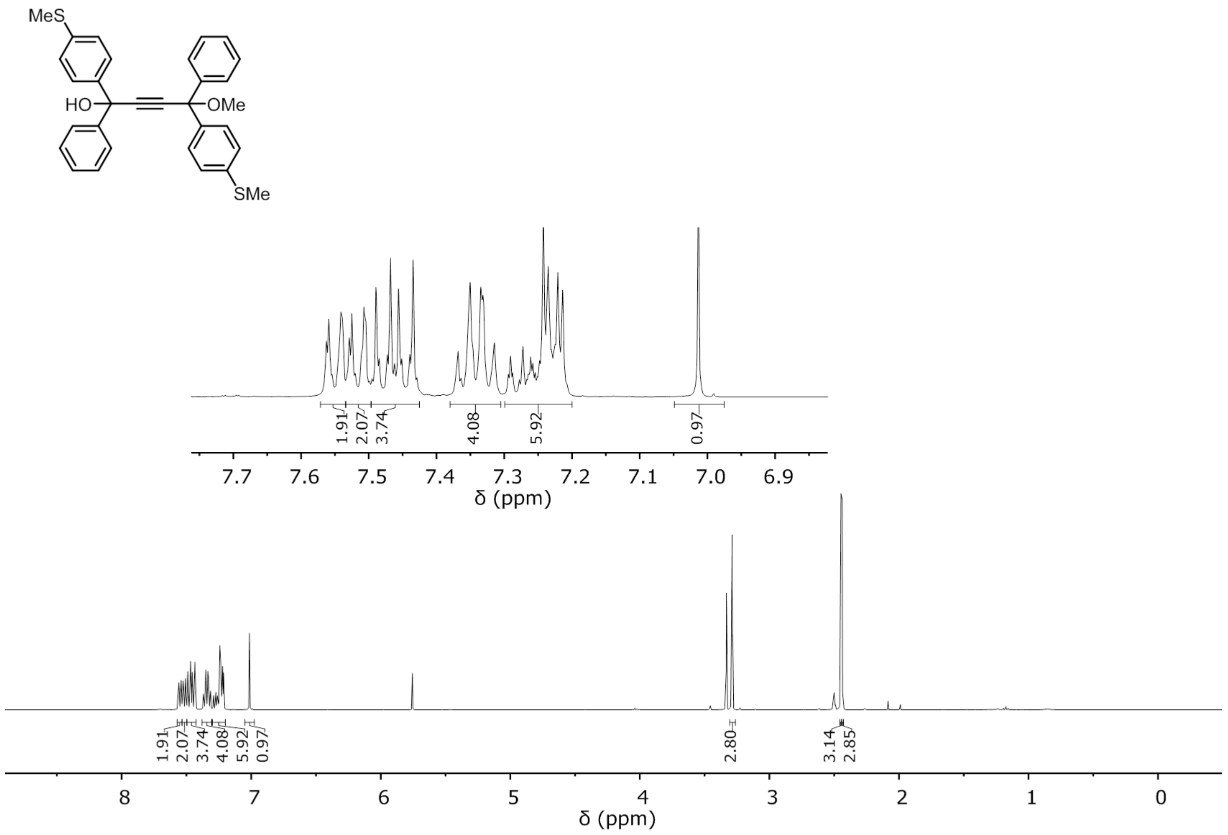

Figure S32. ^{1}H NMR spectrum of 4-methoxy-1,4-bis(4-(methylthio)phenyl)-1,4-diphenylbut-2-yn-1-ol **12** (d_6-DMSO, 400 MHz, 298 K).

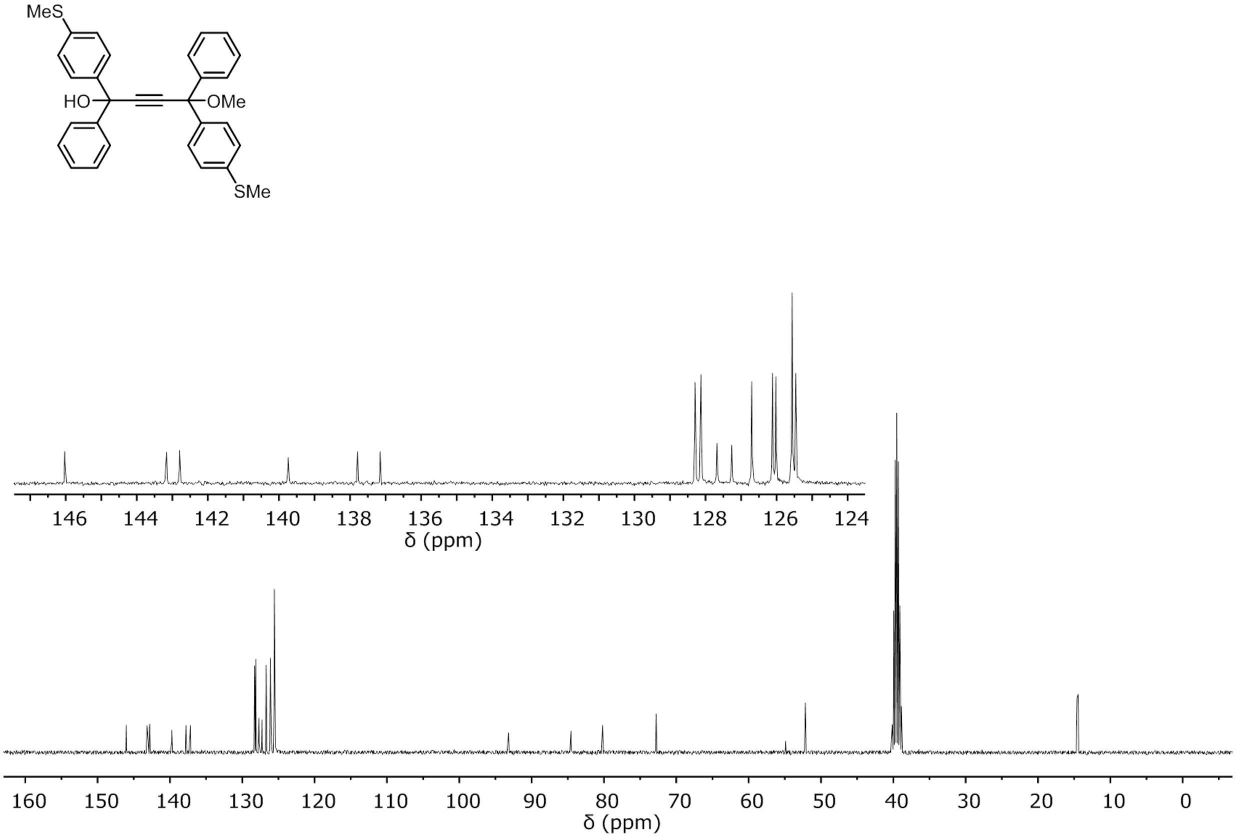

Figure S33. ^{13}C NMR spectrum of 4-methoxy-1,4-bis(4-(methylthio)phenyl)-1,4-diphenylbut-2-yn-1-ol **12** (d_6-DMSO, 100 MHz, 298 K).

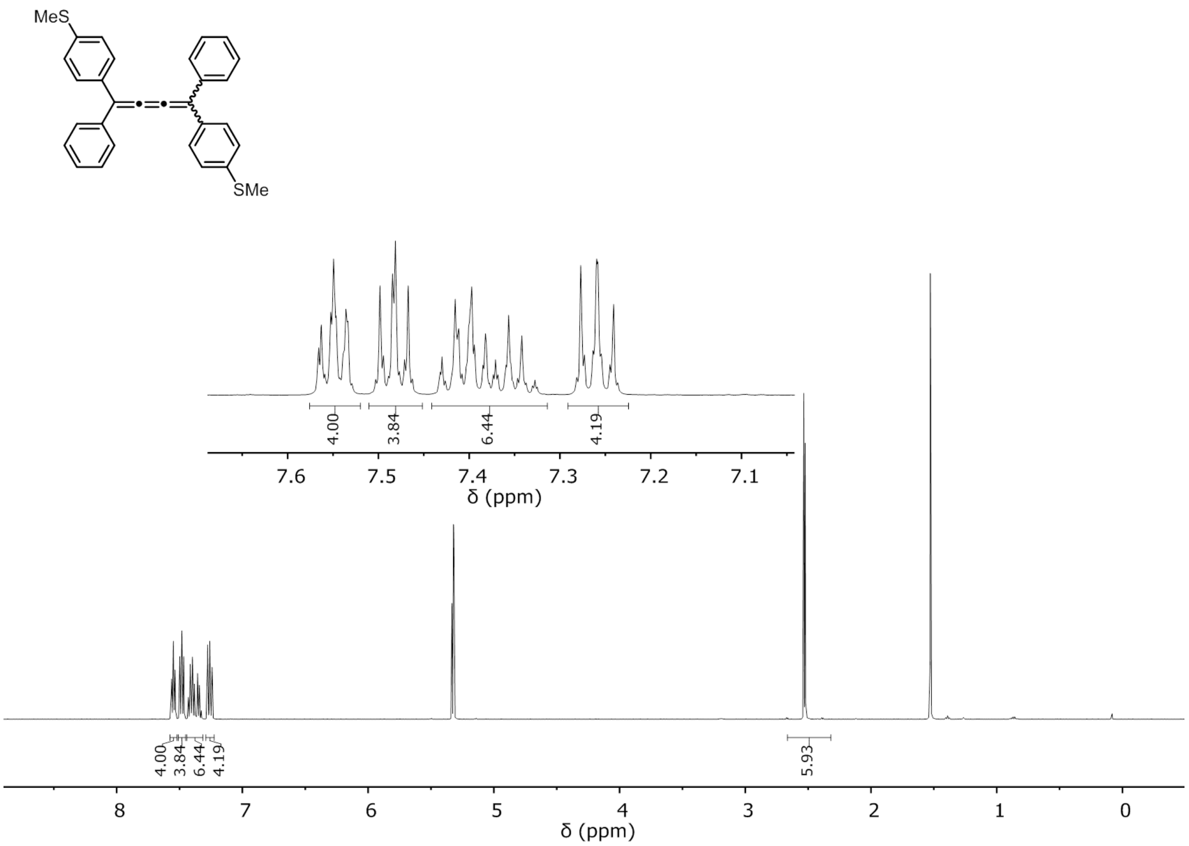

Figure S34. ^{1}H NMR spectrum of 1,4-bis(4-(methylthio)phenyl)-1,4-diphenylbuta-1,2,3-triene **3** (CD_2Cl_2, 500 MHz, 298 K).

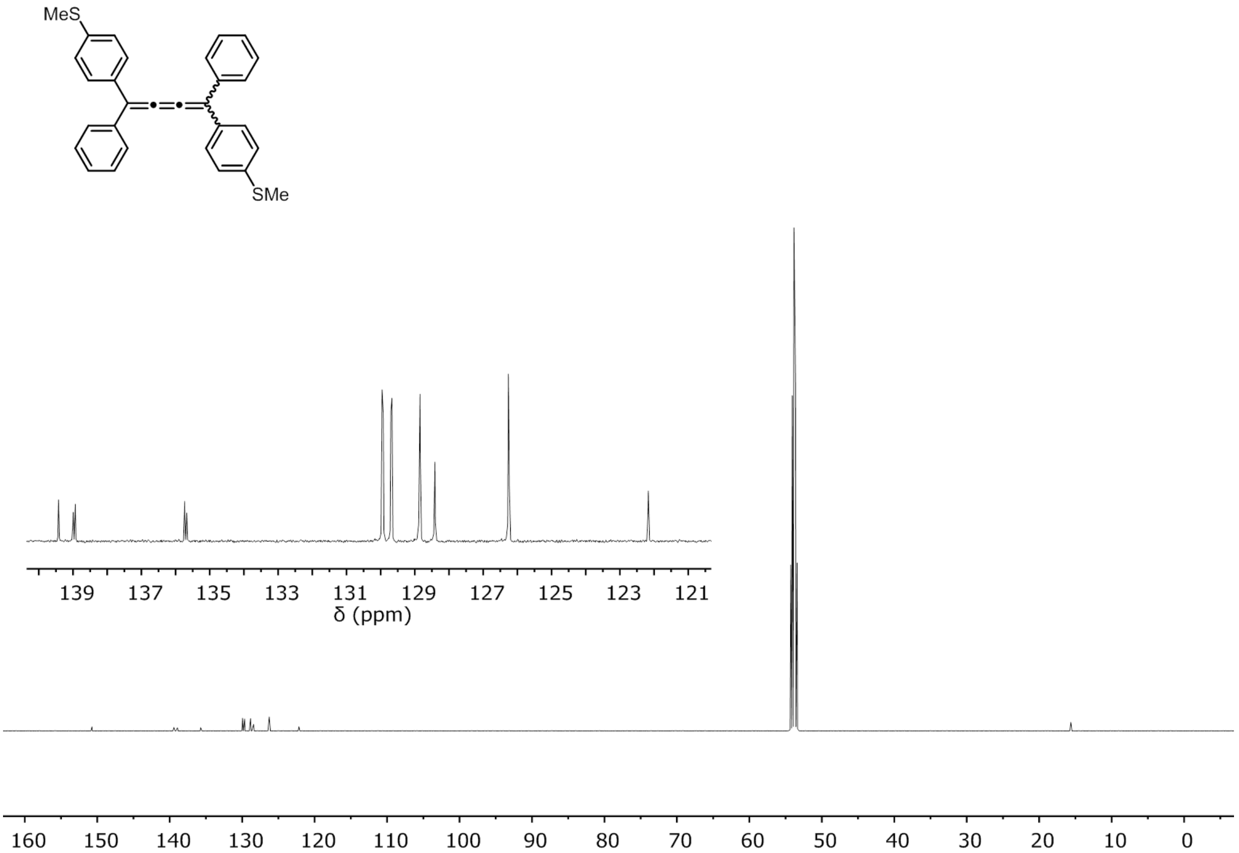

Figure S35. ^{13}C NMR spectrum of 1,4-bis(4-(methylthio)phenyl)-1,4-diphenylbuta-1,2,3-triene **3** (CD_2Cl_2, 125 MHz, 298 K).

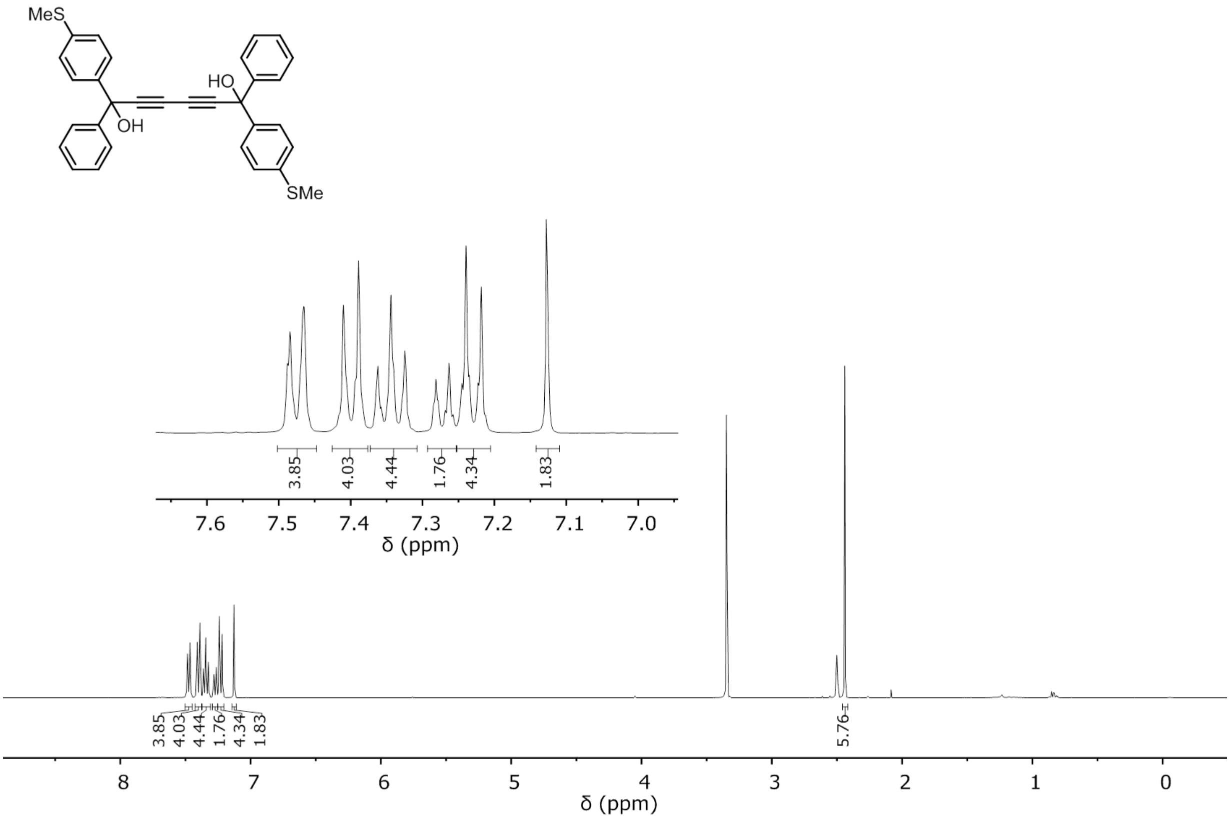

Figure S36. ^{1}H NMR spectrum of 1,6-bis(4-(methylthio)phenyl)-1,6-diphenylhexa-2,4-diyne-1,6-diol **13** (d_6-DMSO, 400 MHz, 298 K).

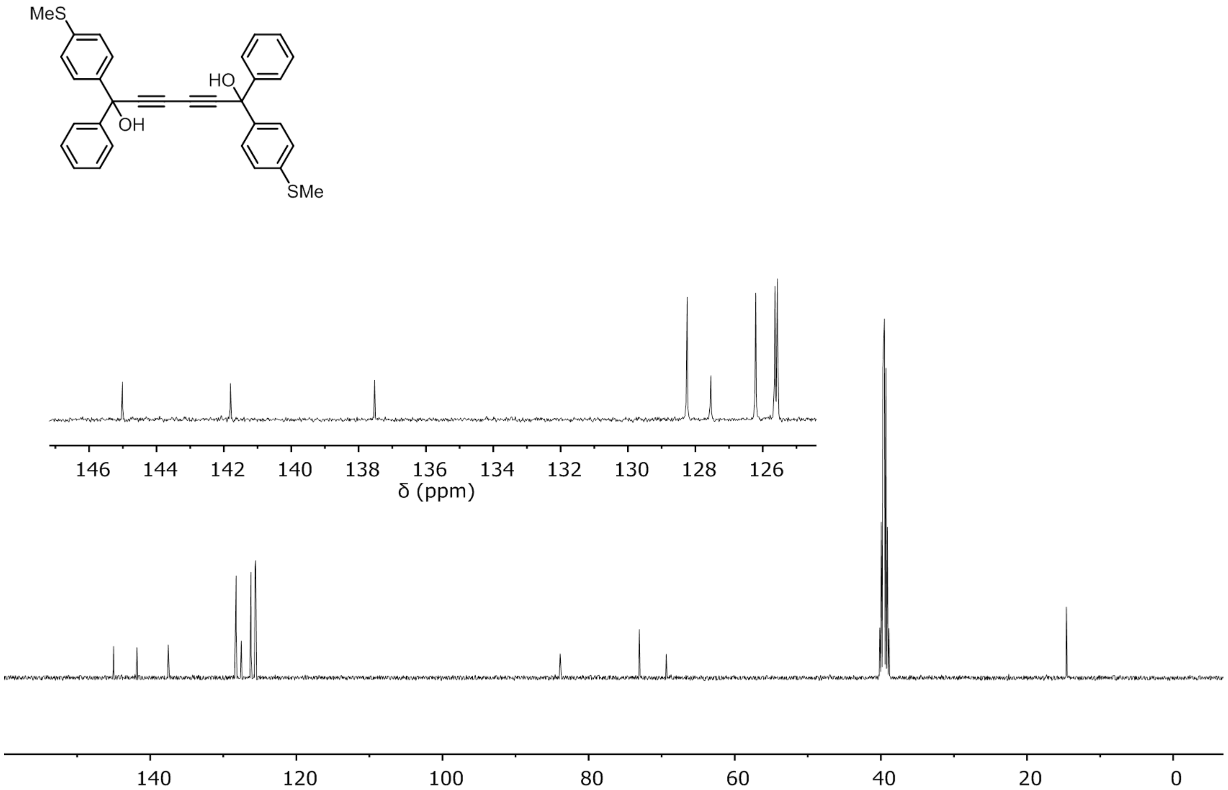

Figure S37. ^{13}C NMR spectrum of 1,6-bis(4-(methylthio)phenyl)-1,6-diphenylhexa-2,4-diyne-1,6-diol **13** (d_6-DMSO, 100 MHz, 298 K).

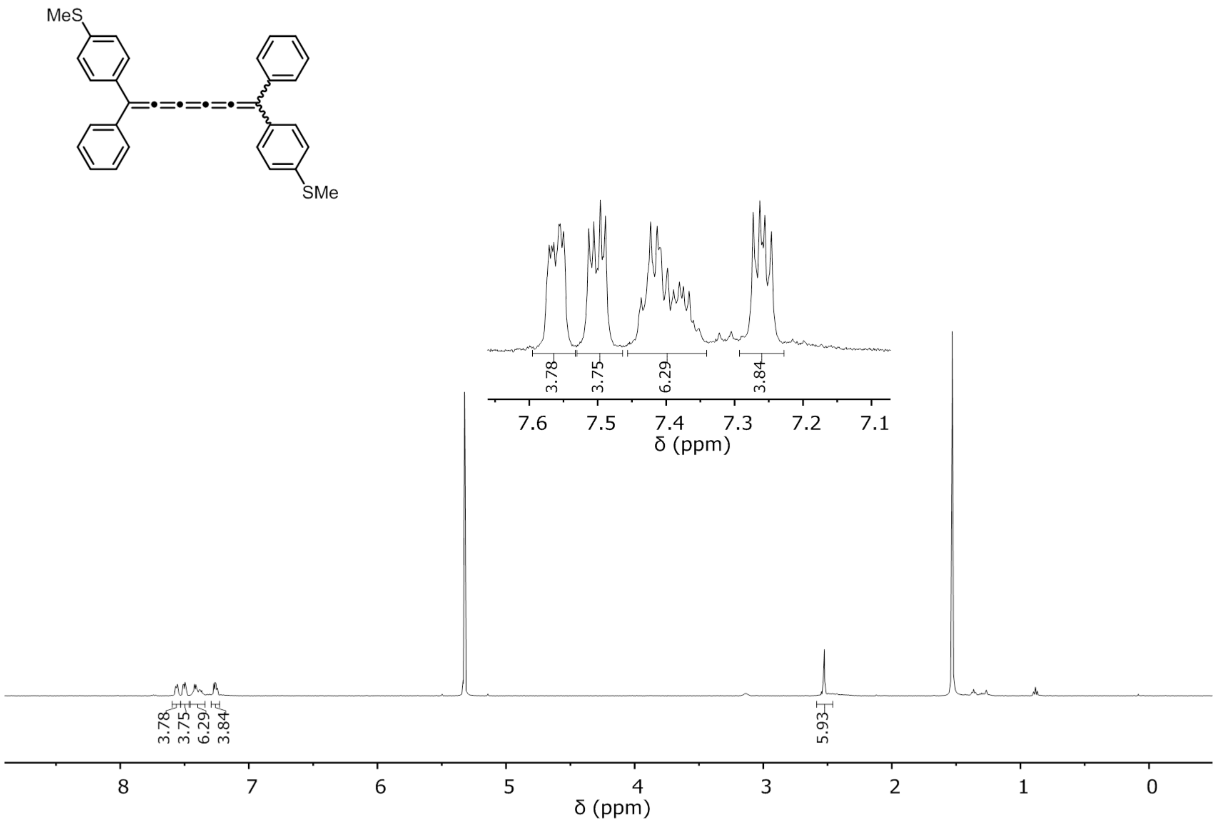

Figure S38. ^{1}H NMR spectrum of 1,6-bis(4-(methylthio)phenyl)-1,6-diphenylhexa-1,2,3,4,5-pentaene **5** (CD_2Cl_2, 500 MHz, 298 K).

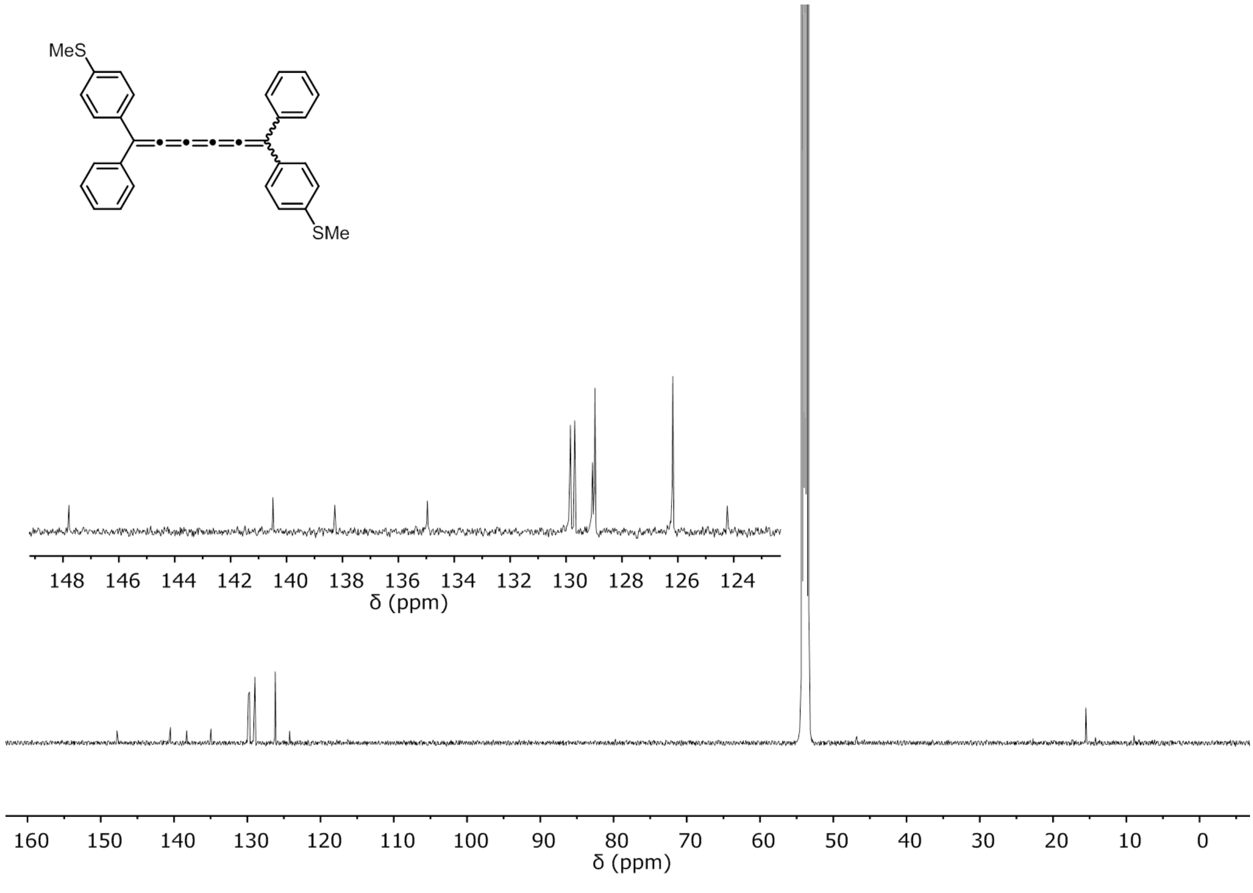

Figure S39. ^{13}C NMR spectrum of 1,6-bis(4-(methylthio)phenyl)-1,6-diphenylhexa-1,2,3,4,5-pentaene **5** (CD_2Cl_2, 125 MHz, 298 K).

Section S5. References

[1] P. Nguyen, Z. Yuan, L. Agocs, G. Lesley, T. B. Marder, *Inorg. Chim. Acta* **1994**, *220*, 289–296.

[2] A. Schoenberg, *Liebigs Ann. Chem.* **1924**, *436*, 205–218.

[3] X. Fang, Y.-M. Zhang, K. Chang, Z. Liu, X. Su, H. Chen, S. X.-A. Zhang, Y. Liu, C. Wu, *Chem. Mater.* **2016**, *28*, 6628–6636.

[4] P. Gawel, C. Dengiz, A. D. Finke, N. Trapp, C. Boudon, J.-P. Gisselbrecht, F. Diederich, *Angew. Chem. Int. Ed.* **2014**, *53*, 4341–4345; *Angew. Chem.* **2014**, *126*, 4430–4434.

[5] L.-M. Wei, L.-L. Wei, W.-B. Pan, M.-J. Wu, *Synlett.* **2005**, *14*, 2219–2223.

[6] N. Islam, T. Ooi, T. Iwasawa, M. Nishiuchi, Y. Kawamura, *Chem. Commun.* **2009**, 574–576.

[7] J. Cosier, A. M. Glazer, *J. Appl. Cryst.* **1986**, *19*, 105–107.

[8] L. Palatinus, G. Chapuis, *J. Appl. Cryst.* **2007**, *40,* 786–790.

[9] a) P. Parois, R. I. Cooper, A. L. Thompson, *Chem. Cent. J.* **2015**, *9*:30; b) R. I. Cooper, A. L. Thompson, D. J. Watkin, *J. Appl. Cryst.* **2010**, *43*, 1100–1107.

[10] J. M. Soler, E. Artacho, J. D. Gale, A. Garcìa, J. Junquera, P. Ordejòn, D. Sànchez-Portal, *J. Phys. Condens. Matter* **2002**, *14*, 2745–2779.

[11] J. Ferrer, C. J. Lambert, V. M. García-Suárez, D. Zs Manrique, D. Visontai, L. Oroszlany, R. Rodríguez-Ferradás, I. Grace, S. W. D. Bailey, K. Gillemot, H. Sadeghi, L. A. Algharagholy, *New J. Phys.* **2014**, *16*, 093029.

[12] V. M. García-Suárez, C. J. Lambert, *Nanotechnology* **2008**, *19*, 455203.

[13] C. Wang, A. S. Batsanov, M. R. Bryce, S. Martín, R. J. Nichols, S. J. Higgins, V. M. García-Suárez, C. J. Lambert, *J. Am. Chem. Soc.* **2009**, *131*, 15647–15654.

[14] P. Moreno-García, M. Gulcur, D. Z. Manrique, T. Pope, W. Hong, V. Kaliginedi, C. Huang, A. S. Batsanov, M. R. Bryce, C. Lambert, T. Wandlowski, *J. Am. Chem. Soc.* **2013**, *135*, 12228−12240.

[15] A. Al-Backri, V. Zólyomi, C. J. Lambert, *J. Chem. Phys.* **2014**, *140*, 104306.

[16] E. Mostaani, B. Monserrat, N. D. Drummonda, C. J. Lambert, *Phys. Chem. Chem. Phys.* **2016**, *18*, 14810–14821.

[17] M. U. Bühringer, K. Padberg, M. D. Phleps, H. Maid, C. Placht, C. Neiss, M. J. Ferguson, A. Görling, R. R. Tykwinski, *Angew. Chem. Int. Ed.* **2018**, *57*, 8321−8325; *Angew. Chem.* **2018**, *130*, 8454–8458.

[18] C. R. Parker, E. Leary, R. Frisenda, Z. Wei, K. S. Jennum, E. Glibstrup, P. Bæch Abrahamsen, M. Santella, M. A. Christensen, E. Antonio Della Pia, T. Li, M. Teresa Gonzalez, X. Jiang, T. J. Morsing, G. Rubio-Bollinger, B. W. Laursen, K. Nørgaard, H. van der Zant, N. Agrait, M. Brøndsted Nielsen, *J. Am. Chem. Soc.* **2014**, *136*, 16497–16507.

[19] M. T. González, X. Zhao, D. Z. Manrique, D. Miguel, E. Leary, M. Gulcur, A. S. Batsanov, G. Rubio-Bollinger, C. J. Lambert, M. R. Bryce, N. Agraït, *J. Phys. Chem. C* **2014**, *118*, 21655−21662.

[20] S. Gunasekaran, D. Hernangómez-Pérez, I. Davydenko, S. Marder, F. Evers, L. Venkataraman, *Nano. Lett.* **2018**, *18*, 6387−6391.

[21] E. Leary, B. Limburg, A. Alanazy, S. Sangtarash, I. Grace, K. Swada, L. J. Esdaile, M. Noori, M. T. González, G. Rubio-Bollinger, H. Sadeghi, A. Hodgson, N. Agraït, S. J. Higgins, C. J. Lambert, H. L. Anderson, R. J. Nichols, *J. Am. Chem. Soc.* **2018**, *140*, 12877−12883.

[22] S. Wu, M. T. González, R. Huber, S. Grunder, M. Mayor, C. Schönenberger, M. Calame, *Nature Nano.* **2008**, *3*, 569–574.

[23] A. Magyarkuti, O. Adak, A. Halbritter, L. Venkataraman, *Nanoscale* **2018**, *10*, 3362–3368.
